# Integrity of Corpus Callosum Is Essential for the Cross-Hemispheric Propagation of Sleep Slow Waves: A High-Density EEG Study in Split-Brain Patients

**DOI:** 10.1523/JNEUROSCI.2571-19.2020

**Published:** 2020-07-15

**Authors:** Giulia Avvenuti, Giacomo Handjaras, Monica Betta, Jacinthe Cataldi, Laura Sophie Imperatori, Simona Lattanzi, Brady A. Riedner, Pietro Pietrini, Emiliano Ricciardi, Giulio Tononi, Francesca Siclari, Gabriele Polonara, Mara Fabri, Mauro Silvestrini, Michele Bellesi, Giulio Bernardi

**Affiliations:** ^1^Molecular Mind Laboratory Research Unit, IMT School for Advanced Studies, 55100 Lucca, Italy; ^2^Center for Investigation and Research on Sleep, Lausanne University Hospital, CH-1011 Lausanne, Switzerland; ^3^Department of Experimental and Clinical Medicine, Marche Polytechnic University, 60121 Ancona, Italy; ^4^Department of Psychiatry, University of Madison, Madison, Wisconsin 53706; ^5^Department of Odontostomatologic and Specialized Clinical Sciences, Marche Polytechnic University, 60121 Ancona, Italy; ^6^School of Physiology, Pharmacology and Neuroscience, University of Bristol, Bristol BS8 1TD, United Kingdom

**Keywords:** connectivity, corpus callosum, NREM, sleep, slow wave

## Abstract

The slow waves of non-rapid eye movement (NREM) sleep reflect experience-dependent plasticity and play a direct role in the restorative functions of sleep. Importantly, slow waves behave as traveling waves, and their propagation is assumed to occur through cortico-cortical white matter connections. In this light, the corpus callosum (CC) may represent the main responsible for cross-hemispheric slow-wave propagation. To verify this hypothesis, we performed overnight high-density (hd)-EEG recordings in five patients who underwent total callosotomy due to drug-resistant epilepsy (CPs; two females), in three noncallosotomized neurologic patients (NPs; two females), and in a sample of 24 healthy adult subjects (HSs; 13 females). In all CPs slow waves displayed a significantly reduced probability of cross-hemispheric propagation and a stronger inter-hemispheric asymmetry. In both CPs and HSs, the incidence of large slow waves within individual NREM epochs tended to differ across hemispheres, with a relative overall predominance of the right over the left hemisphere. The absolute magnitude of this asymmetry was greater in CPs relative to HSs. However, the CC resection had no significant effects on the distribution of slow-wave origin probability across hemispheres. The present results indicate that CC integrity is essential for the cross-hemispheric traveling of slow waves in human sleep, which is in line with the assumption of a direct relationship between white matter integrity and slow-wave propagation. Our findings also revealed a residual cross-hemispheric slow-wave propagation that may rely on alternative pathways, including cortico-subcortico-cortical loops. Finally, these data indicate that the lack of the CC does not lead to differences in slow-wave generation across brain hemispheres.

**SIGNIFICANCE STATEMENT** The slow waves of NREM sleep behave as traveling waves, and their propagation has been suggested to reflect the integrity of white matter cortico-cortical connections. To directly assess this hypothesis, here we investigated the role of the corpus callosum in the cortical spreading of NREM slow waves through the study of a rare population of totally callosotomized patients. Our results demonstrate a causal role of the corpus callosum in the cross-hemispheric traveling of sleep slow waves. Additionally, we found that callosotomy does not affect the relative tendency of each hemisphere at generating slow waves. Incidentally, we also found that slow waves tend to originate more often in the right than in the left hemisphere in both callosotomized and healthy adult individuals.

## Introduction

The transition from wakefulness to sleep is marked by profound changes in brain EEG activity, with a shift from the low-amplitude, high-frequency signals recorded in wakefulness to the high-amplitude, low-frequency slow waves (0.5–4 Hz) of non-rapid eye movement (NREM) sleep. In particular, the sleep slow wave represents the EEG signature of a slow oscillation in membrane potential at neuronal level, characterized by an alternation between a hyperpolarized “silent” phase (*down-state*) and a depolarized phase of intense firing activity (*up-state*; Steriade et al., 2001). Crucially, the amount of slow-wave activity (SWA; expressed as the 0.5–4 Hz EEG signal power in NREM sleep) represents a reliable marker of homeostatically regulated sleep need (Achermann and Borbély, 2003) and has been shown to be locally modulated in a use-dependent manner, thus implying a possible relationship with plasticity-related processes (Tononi and Cirelli, 2014). Indeed, experimental studies and computer simulations have demonstrated that not only does SWA reflect experience-dependent changes in regional synaptic density/strength, but slow waves may also play a direct role in cellular and systems restoration and in the consolidation of newly acquired memories (Tononi and Cirelli, 2014). Recent evidence also suggested a possible implication of sleep slow waves in the clearance of neurotoxic metabolic products that accumulate during wakefulness (Xie et al., 2013; Hablitz et al., 2019).

The sleep slow waves are not stationary events. Instead, they typically behave as traveling waves at the macroscale level of the scalp EEG, with variable cortical origins and propagation patterns (Massimini et al., 2004; Murphy et al., 2009). Such a propagation is commonly assumed to reflect the structural properties of cortico-cortical white matter connections. In line with this, structural white matter properties have been found to correlate with parameters reflecting slow-wave synchronization (Murphy et al., 2009; Buchmann et al., 2011; Piantoni et al., 2013). In this perspective, the corpus callosum (CC) would be expected to represent the main route responsible for cross-hemispheric slow-wave propagation. However, correlational studies and research in human models of inter-hemispheric disconnection produced contradictory findings. For instance, two studies found a positive significant correlation between macrostructural (volume) and microstructural (axial diffusivity) properties of the CC and parameters reflecting slow-wave synchronization (i.e., amplitude and slope) in healthy adult individuals (Buchmann et al., 2011; Piantoni et al., 2013). In contrast, a more recent work failed to replicate the correlation between slow-wave slope and axial diffusivity in healthy adult subjects, and rather described a positive correlation between indices reflecting white matter damage and slow-wave synchronization in patients with traumatic brain injury (TBI; Sanchez et al., 2019). In addition, while studies performed in patients with agenesis of the CC (Kuks et al., 1987; Nielsen et al., 1993) or in epileptic patients who underwent partial or total callosotomy (Montplaisir et al., 1990) showed a decreased inter-hemispheric coherence within the delta range (<4 Hz) during NREM sleep, callosotomized patients continue to present a clear increase in inter-hemispheric coherence from wakefulness to sleep (Corsi-Cabrera et al., 2006).

**Figure 1.**
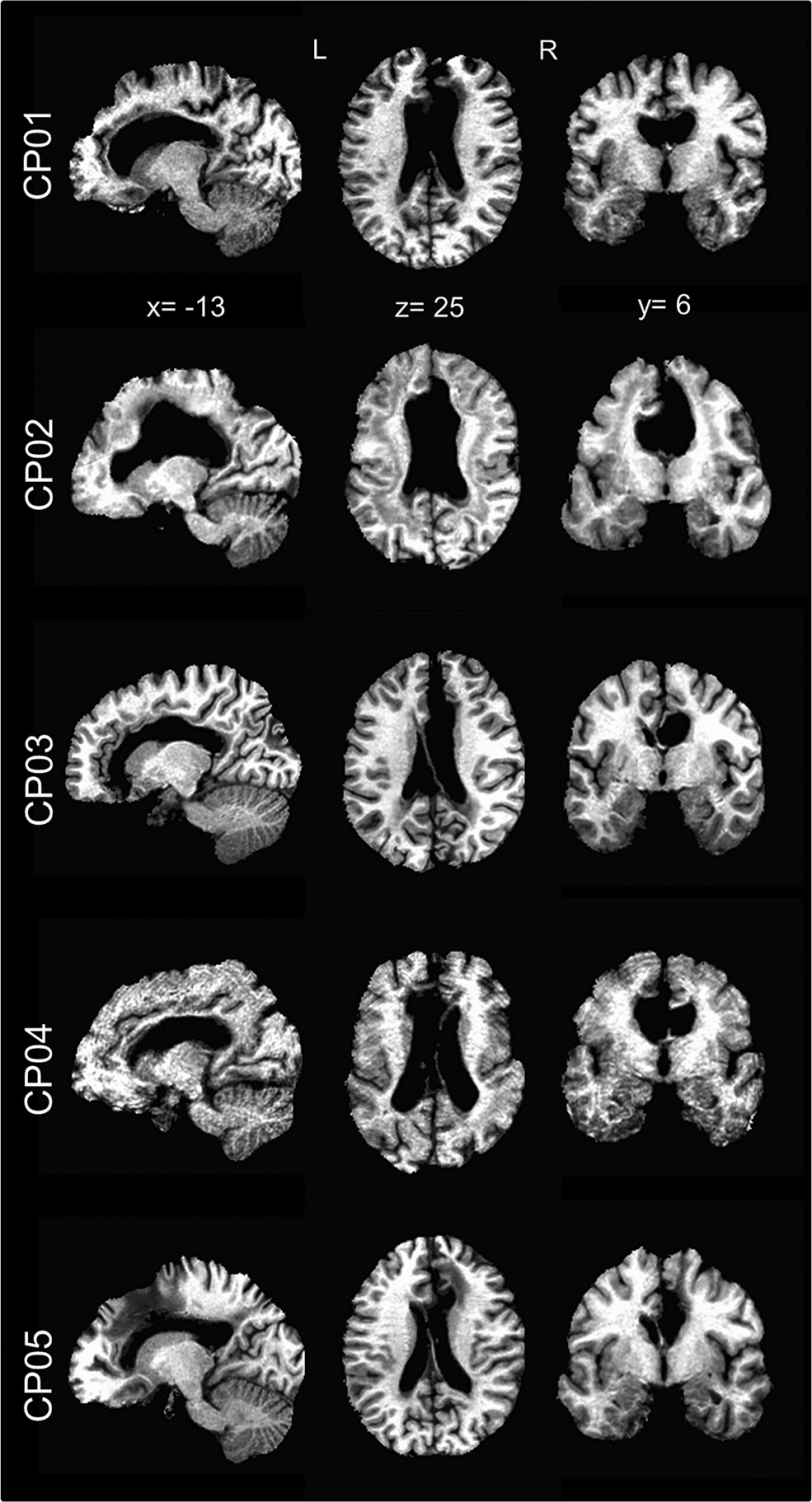
Anatomical MRI images of callosotomized patients. For each patient (CP01–CP05) sagittal, axial, and coronal MRI images are shown in MNI space. It is possible to appreciate the complete absence of the corpus callosum in all cases.

Given the above considerations, it is still unclear how the agenesis or the complete resection of the corpus callosum may affect sleep slow-wave propagation in humans. Crucially, this matter has more general implications for the hypothesized relationship between brain structural connectivity and slow-wave propagation (Murphy et al., 2009), as well as for the understanding of the mechanisms that regulate slow-wave synchronization in relation to plastic and developmental processes (Mascetti et al., 2013; Kurth et al., 2017). Moreover, the contradictory findings reported in the literature likely result mostly from methodological limitations and discrepancies. Therefore, to determine the role of inter-hemispheric white matter connections in slow-wave generation and propagation, here we analyzed for the first time overnight high-density (hd)-EEG recordings (256 electrodes) collected in a sample of five patients with epilepsy who underwent total callosotomy (CPs; Fig. 1) and in control subjects with an intact CC, including three neurological noncallosotomized patients (NPs, one male with epilepsy) and 24 healthy adult subjects (HSs). To overcome the limitations of previous studies related to the use of indirect indices of slow-wave synchronization and propagation, we used validated algorithms to detect individual slow waves and to determine their specific origin and traveling pattern.

## Materials and Methods

### Participants

Overnight hd-EEG recordings (256 electrodes; EGI-Philips) were performed at the Neurologic Unit of the Marche Polytechnic University (Ancona, Italy) in five inpatients with epilepsy who underwent a total resection of the CC (i.e., CPs; age range, 40–53 years; two females; Fig. 1). Three noncallosotomized neurologic inpatients (NPs, age range, 44–66 years; two females) were also studied under the same experimental conditions. Symptomatic generalized epilepsy due to viral meningoencephalitis occurring in infancy was diagnosed in one of these patients (this subject, indicated as NP03, was marked using a distinctive color in figures). All of the noncallosotomized patients had no diagnoses of any other comorbidities affecting brain function at the time of the study. Tables 1 and 2 report demographic and clinical characteristics for all patients. An additional control group of 24 healthy adult volunteers (i.e., HSs; age range, 20–47 years; 13 females) was studied with the same hd-EEG recording system at the Lausanne University Hospital (Lausanne, Switzerland; analyses of NREM sleep data from these subjects, not involving the study of inter-hemispheric slow-wave propagation, have been reported in previous work; Siclari et al., 2018; Bernardi et al., 2019a,b). Before their inclusion in the study, HS group individuals underwent a clinical interview to exclude a history of sleep, medical, and psychiatric disorders. None of the HSs was taking any medication at the time of the study. The study procedures were conducted under clinical research protocols approved by the local ethical committees and in accordance with the guidelines of the Declaration of Helsinki. Written informed consent was obtained from all participants.

**Table 1.** Demographic and clinical characteristics

	CP01	CP02	CP03	CP04	CP05	NP01	NP02	NP03	HS (*n* = 24)
Age, years	53	40	47	45	42	45	66	44	27 ± 6
Gender	M	F	F	M	M	F	F	M	13F
Age at surgery 1, years	30	16	25	14	18				
Age at surgery 2, years	45	17	26	22	19				
Questionnaires									
PSQI	12	2	4	5	9	11	4	12	3.1 ± 1.5
ESS	23	4	2	0	13	7	18	1	5.9 ± 2.1
HOQ	59	44	43	61	54	52	34	45	51.8 ± 6.7
EHI	RH	RH	RH	RH	RH	RH	RH	RH	20RH
Sleep structure									
Sleep latency, min	20.5	29.5	23.5	38.5	34.0	6.0	6.5	4.0	15.4 ± 16.9
Total sleep time, min	244.5	243.5	185.5	254.0	120.5	278.0	251.5	118.5	256.6 ± 30.6
Sleep efficiency, %	81.4	81.0	61.7	84.5	40.1	92.5	83.7	39.4	85.5 ± 10.2
WASO, min	36	15.5	66.0	4.5	129.5	11.0	24.5	157.5	20.0 ± 15.3
N1 sleep, %	0.8	7.0	14.8	9.6	24.5	3.1	8.5	19.4	5.1 ± 5.0
N2 sleep, %					80.9	63.5	66.0	59.1	57.7 ± 10.4
N3 sleep, %					19.1	9.2	15.7	19.4	27.1 ± 7.5
NREM (N2/N3) sleep, %	100.0	88.7	100.0	100.0	100.0	72.7	81.7	78.5	84.8 ± 6.0
REM sleep, %	0.0	11.3	0.0	0.0	0.0	27.3	18.3	21.5	15.2 ± 6.0

For patients in the CP and NP groups, demographic characteristics, questionnaires, and sleep macroarchitecture are presented separately for each subject. For CP patients, the resection of the corpus callosum occurred in two distinct surgical interventions, for each of which the age of the patients is reported. For the HS group, the values are reported as the group mean ± SD. Sleep stage percentages are expressed with respect to total sleep time (TST). PSQI, Pittsburgh Sleep Quality Index; ESS, Epworth Sleepiness Scale; HOQ, Horne-Őstberg Questionnaire; EHI, Edinburgh Handedness Inventory; PSG, polysomnography; WASO, wake after sleep onset.

**Table 2.** Clinical diagnosis and medications of studied patients

	Diagnosed pathology	Current medications
CP01	Lennox-Gastaut syndrome	Carbamazepine, phenytoin sodium, phenobarbital
CP02	Drug-resistant epilepsy	Carbamazepine, levetiracetam, sodium valproate
CP03	Early infantile epileptic encephalopathy	Levosulpiride, oxcarbazepine, phenobarbital, risperidone
CP04	Lennox-Gastaut syndrome	Clobazam, lacosamide, phenobarbital, rosuvastatin, vigabatrin
CP05	Drug-resistant epilepsy	Carbamazepine, clonazepam, diazepam, omeprazole, phenobarbital
NP01	Generalized anxiety disorder	None
NP02	Lumbar spinal stenosis	Cholecalciferol, esomeprazole, lisinopril, mometasone
NP03	Epilepsy (viral meningoencephalitis in infancy)	Enalapril + lercanidipine, lacosamide, oxcarbazepine, sodium valproate, Ursodeoxycholic acid

Clinical diagnosis and medications of studied patients.

### Data acquisition

One overnight hd-EEG recording (500 Hz sampling rate) was obtained for each subject. All recordings were initiated at the usual bedtime of each participant and interrupted at approximately 7:00 A.M. Given that callosotomized patients had a relatively low sleep quality with frequent awakenings especially in the second part of the night, we extracted and analyzed only the first 5 h of each recording, starting from the time of “lights-off.” To ensure across-group comparability, analyses similarly focused only on the first 5 h of data also in the healthy control subjects. Three 2 min “resting-state” hd-EEG recordings (6 min in total) were also collected during relaxed wakefulness with the eyes closed before sleep and in the morning, ∼40 min after awakening.

### Data preprocessing

For all patients, recordings were band-pass filtered between 0.1 and 45 Hz. Then, overnight recordings were divided into 30 s epochs, while wake resting-state recordings were divided into 4 s epochs. Bad channels and epochs were identified and rejected through visual inspection in NetStation 5.3 (EGI-Philips). An independent component analysis (ICA) procedure was used to reduce residual ocular, muscular, and electrocardiographic artifacts (EEGLAB toolbox; Delorme and Makeig, 2004). Finally, rejected bad channels were interpolated using spherical splines. A similar procedure was used to preprocess the sleep data of healthy control subjects, as described in previous work (Bernardi et al., 2019b). Data were filtered between 0.5 and 40 Hz before the analyses of signal power and slow-wave parameters.

### Sleep scoring

**Figure 2.**
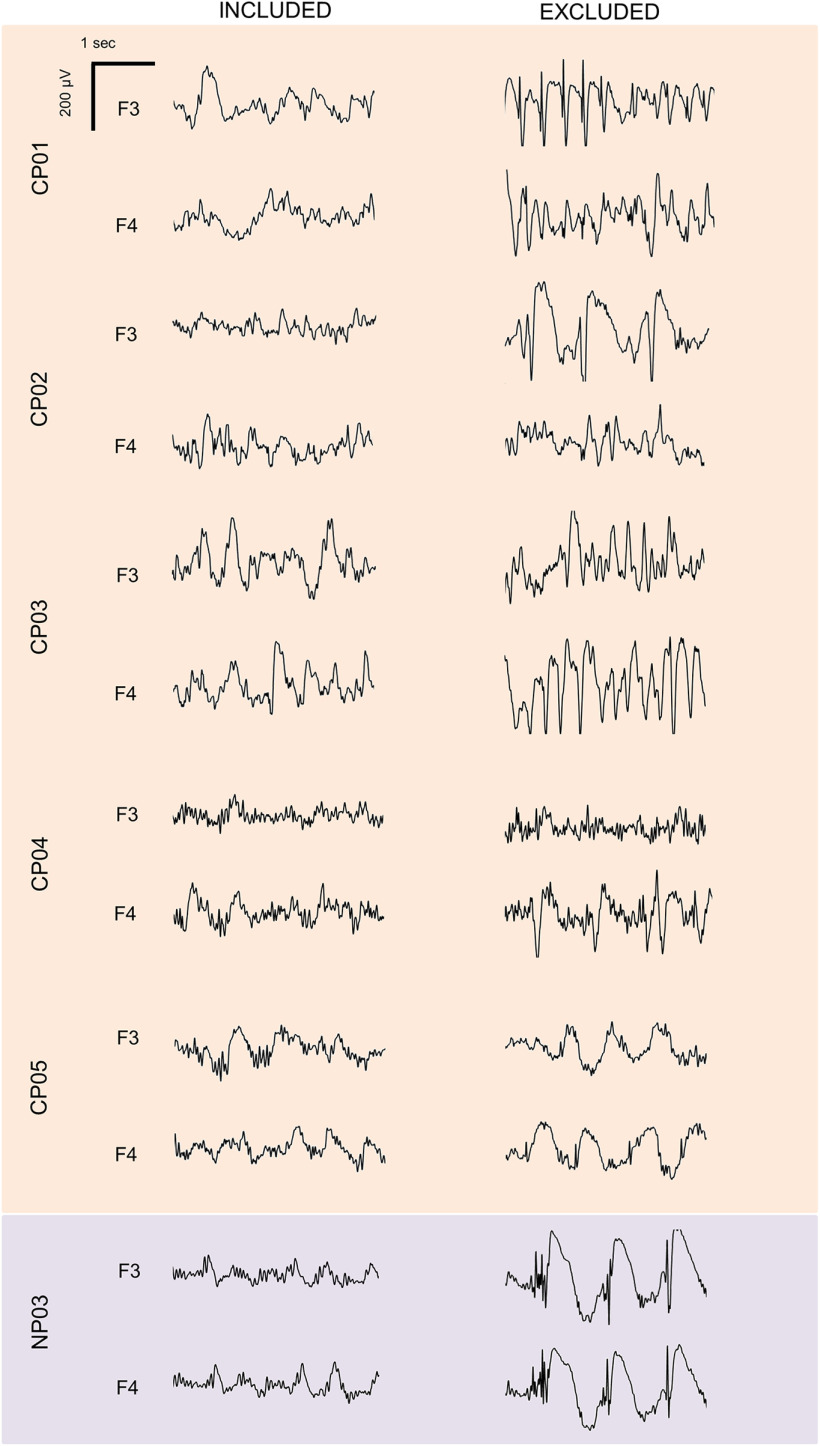
Examples of included and excluded data segments in patients with epilepsy. For each subject, representative EEG traces (0.5–25 Hz) corresponding to included (left) and excluded (right) data segments are shown for one frontal left (F3) and one frontal right (F4) electrode.

For scoring purposes, four electrodes were used to monitor horizontal andvertical eye movements (electrooculography), while electrodes located in thechin-cheek region were used to evaluate muscular activity (electromyography). Sleep scoring was performed over 30 s epochs according to the criteria from the American Academy of Sleep Medicine scoring manual (Iber et al., 2007). Two operators took care in marking periods containing large artifacts, arousals, and nonphysiological activity (Fig. 2, examples of included and excluded data for epilepsy patients). Only slow waves detected within artifact-free NREM sleep (N2/N3) data segments were analyzed.

### Power computation

A current–source density (CSD) transform was applied to all EEG recordings using the CSD toolbox (Kayser and Tenke, 2006). This method provides a reference-independent signal and improves spatial resolution by acting as a spatial filter. For each EEG derivation, power spectral density (PSD) estimates were computed using Welch's method in 4 s data segments (Hamming windows, 8 sections, 50% overlap) and integrated within the delta/SWA (0.5–4 Hz) and the beta (18–35 Hz) frequency bands. In all sleep epochs, the power computation was performed over seven 4 s segments (28 s)after excluding the first and the last second of data.

### Slow-wave detection

Slow waves were detected automatically in a composite EEG signal generated from linked-mastoid referenced channels, as previously described (Siclari et al., 2014; Mensen et al., 2016; Bernardi et al., 2018). This method provides a unique time reference (across electrodes) for each slow wave and facilitates the detection of both local and widespread events (Mensen et al., 2016). Specifically, a negative-going signal envelope was calculated by selecting the fifth most negative sample across a subset of 191 electrodes obtained by excluding channels located on the neck and face regions. This approach minimizes the risk of including in the envelope potential residual high-amplitude oscillations of artifactual origin. Finally, the obtained signal envelope was broadband filtered (0.5–40 Hz) before the application of a slow-wave detection procedure based on half-wave zero-crossings (Vyazovskiy et al., 2007; Siclari et al., 2014). Only half-waves with a duration of between 0.25 and 1.0 s were retained for further analyses. Of note, no amplitude thresholds were applied based on previous evidence indicating the following: (1) slow waves with peak-to-peak amplitude <75 µV show the clear homeostatic changes commonly attributed to the slow waves of NREM sleep (Vyazovskiy et al., 2007; Bernardi et al., 2018); and (2) the application of an amplitude threshold may actually preferentially select a minority of very large slow waves that have been shown to display different regulation and synchronization mechanisms compared with the majority of slow waves (Mensen et al., 2016; Siclari et al., 2014, 2018; Spiess et al., 2018; Bernardi et al., 2018, 2019a). For all the detected slow waves, various parameters of interest were calculated and stored for a subsequent evaluation, including negative amplitude (in microvolts), descending slope (between the first zero-crossing and the maximum negative peak; in microvolts per millisecond) and involvement (mean EEG-signal calculated for all electrodes in an 80 ms window centered on the wave peak; in microvolts). Moreover, the slow-wave density (the number of waves per minute) was computed in each sleep epoch (epochs in which artifactual or nonphysiological activity occupied >75% of the time were excluded).

### Scalp involvement distribution

For each subject, the involvement distribution (across channels) of all slow waves was analyzed using principal component analysis (PCA), as described in previous work (Bernardi et al., 2018). We recently showed that in healthy adult subjects the 95% of the variance related to slow-wave involvement is explained by three principal components (PCs), with maxima located in the centrofrontal area (∼70% of total variance), anterior or posterior areas (∼20%), and left or right hemispheres (∼5%). Here we hypothesized that callosotomized patients would present an increased variance explained by the last, unihemispheric PC at the expenses of the other two, symmetrical, components. To test this hypothesis, we first verified through visual inspection that similar PCs explaining a similar amount of total variance were present in HSs (95.0 ± 1.5%; range, 92.3–97.0%), NPs (96.6 ± 0.6%; range, 96.2–97.3%; relative to HS, all *p*_uncorrected_ > 0.099, |*z*| < 1.652) and CPs (94.6 ± 1.3%; range, 93.1–95.9%; all *p*_uncorrected_ > 0.193, |*z*| < 1.302) subjects. Then, the PC-space of each subject was rotated into a common, reference PC-space using the Procrustes transformation (Schönemann, 1966; Haxby et al., 2011). The Procrustes transformation is an orthogonal transformation that minimizes the Euclidean distance between two sets of paired vectors. The reference space was selected by iteratively applying the transformation over pairs of subjects of the HS group and then identifying the coordinate system (i.e., the subject) presenting the smallest distance with respect to the coordinate systems of all tested subjects (Haxby et al., 2011). Finally, the Procrustes transformation was applied to remap the original PC-space of each subject (including the patients), into the new reference PC-space. This procedure allowed us to compare directly the first three PCs (and their explained variances) across individuals.

### Slow-wave propagation

For each detected slow wave, the pattern of propagation was calculated by determining the topographic distribution of relative delays in the local maximum negative peak, representing the moment of maximal regional recruitment (Massimini et al., 2004). To minimize the impact of potential localized artefacts, a “likeness constraint” method (Menicucci et al., 2009) was used to discard channels in which the negative wave was excessively dissimilar from a “prototype” slow wave, defined as the wave with the largest negative peak at the reference peak timing across all channels. Specifically, we calculated the cross-correlation between the instantaneous phases (estimated using the Hilbert transform) of the prototype wave and of all other EEG signals in a symmetrical 300 ms time-window centered on the reference peak. The 25th percentile of the distribution of the maximal cross-correlation values (C) was then used as a threshold to exclude events dissimilar from the prototype wave. The latency of all remaining local peaks was subsequently used to create a preliminary scalp “delay map.” A spatiotemporal clusterization procedure was applied to exclude potential propagation gaps, as follows: local peaks of two spatial neighbor electrodes had to be separated by <10 ms to be considered as part of the same propagation cluster. This approach ensures that all the electrodes are connected to one another through some neighboring electrodes, thus eliminating islands of channels that are likely to reflect artifacts in the local EEG signal. Then, the propagation cluster including the prototype wave was identified and the final delay map was extracted. The minimum delay, corresponding to the slow-wave origin, was set to zero. Finally, a three-dimensional gradient (two for direction, one for timing) was computed from the delay map to identify the main streamlines of propagation for each slow wave. Up to three streamlines were extracted: the longest displacement (the distance between the start and end points of the wave); the longest distance traveled (the cumulative sum of all coordinates of the line); and the stream of the most angular deviation from the longest displacement (the minimum trajectory difference was set to 45°). The streamline corresponding to the longest distance traveled was used to compute the slow-wave speed (distance divided by maximum delay; in meters per second).

### Cross-hemispheric propagation

Information obtained from the propagation analysis was used to compute parameters reflecting the degree of cross-hemispheric propagation of sleep slow waves. First, for each slow wave we determined whether at least one of the propagation streamlines passed the midline (nasion–inion axis). This information was used to compute the proportion of cross-hemispheric slow waves in each subject (percentage of all of the detected slow waves). Second, we determined the relative distribution of electrodes involved in the same (propagating) slow wave across the two hemispheres. This information was used to compute an index of channel recruitment asymmetry, defined as the number of channels in the hemisphere with less involved electrodes, divided by the total number of involved channels (percentage). In this index, a value of 50% indicates a symmetric distribution, while a value of 0% indicates a unilateral wave. This second parameter was also computed for slow waves subdivided into the following five amplitude percentile classes: 0–20, 20–40, 40–60, 60–80, and 80–100.

### Inter-hemispheric differences in slow-wave latency

A resection of the CC may not completely abolish the cross-hemispheric spreading of sleep slow waves. Theoretically, an apparent hemispheric synchronization may result from volume conduction of electrophysiological signals, while a real spreading could occur through alternative pathways involving subcortical structures. To test these hypotheses, we analyzed the relative time-lag between homologous symmetrical electrodes in the two brain hemispheres. Unlike the analyses described above, this investigation was performed using a more conventional slow-wave detection approach, in which negative half-waves were automatically identified in a subset of EEG electrodes, as described in previous work (Mölle et al., 2002; Riedner et al., 2007). Specifically, linked-mastoid-referenced EEG signals of electrodes corresponding to F3 (frontal left) and F4 (frontal right) were filtered between 0.5 and 4 Hz, and negative half-waves with a duration between 0.25 and 1.0 s were detected and retained for further analyses. For each slow wave with a peak-to-peak amplitude of 75 µV detected in F3, we evaluated whether another negative wave of any amplitude was present in F4 within a 140 ms time window centered on the negative peak of the F3 slow wave. Of note, this window length has been selected as it approximately corresponds to the time interval necessary for slow waves to travel from one electrode to the other, assuming a minimum propagation speed of 1 m/s (Massimini et al., 2004). In addition, the amplitude threshold of 75 µV was selected as it represents a criterion commonly adopted in the clinical practice for the definition of sleep slow waves. To verify the consistency of this analysis with evaluations of cross-hemispheric slow-wave propagation described above, we computed in each subject the proportion of bilateral slow waves with respect to all slow waves detected in F3. Moreover, for all bilateral slow waves we determined the absolute time-lag between their negative and positive peaks (respectively, the maximum negative peak between the positive-to-negative and the negative-to-positive zero-crossings, and the maximum positive peak after the negative-to-positivezero-crossing and before the following zero-crossing). Finally, we also determined the proportion of bilateral slow waves showing a zero-lag time difference, which could result from volume conduction rather than a true inter-hemispheric spreading. Of note, similar results were obtained using F4 as a reference channel, or using central (C3, C4) instead of frontal electrodes (data not shown).

### Inter-hemispheric differences in slow-wave density

The CC could be involved not only in the propagation of individual slow waves but also in homogenizing sleep depth across the two hemispheres. In other words, the lack of inter-hemispheric connections could lead to the emergence of hemispheric asymmetries in the relative density of large slow waves, potentially even to a marked unihemispheric sleep. To test this hypothesis, slow waves were automatically detected using the conventional approach described above in three left (F3, C3, P3) and three right (F4, C4, P4) homologous electrodes (Vyazovskiy et al., 2007). A peak-to-peak amplitude threshold of 75 µV was applied; similar results were also obtained using a 40 µV negative-amplitude threshold. The density of slow waves in each epoch and channel was computed as described above. Finally, the average absolute inter-hemispheric difference in slow-wave density was computed across pairs of homologous electrodes.

### Probabilistic origin and recruitment

Next, we evaluated whether slow waves originate with a different incidence across the two hemispheres. Thus, individual slow waves were classified as having a left hemisphere (right hemisphere) origin if >75% of the origin channels (delay = 0 ms) were located in the left (right) hemisphere. An origin asymmetry index was computed as the difference in the density (in waves per minute) of slow waves originating in the left versus the right hemisphere. In addition, we defined the slow-wave “probabilistic origin” as the probability for each channel to represent the origin of a slow wave, computed with respect to all the detected slow waves. Similarly, the “probabilistic recruitment” was defined as the probability for each electrode to be part of the propagation path of a slow wave.

### Statistical analyses

For each parameter of interest, the five CPs and the three NPs (Ancona dataset) were compared with the control group of HSs (Lausanne dataset). Specifically, for each patient, the relative *z* score and corresponding *p* value were computed with respect to the distribution represented by the healthy control group. A Bonferroni correction was applied to account for multiple comparisons across tested subjects and related hypotheses. Effects were regarded as significant only when a corrected value of *p* < 0.05 was observed in each of the five CPs and in none of the three NPs (Table 3 summarizes group-level and subject-level statistics for each performed comparison). Analyses were repeated after regression-based adjustment of values to account for inter-subject age differences. For analyses performed in individual groups (HS, CP) against the null hypothesis of no inter-hemispheric asymmetry, a bootstrapping procedure (1000 iterations) was applied to compute confidence intervals (bCIs; α = 0.05).

**Table 3.** Summary of statistics related to comparisons between patients and healthy subjects

Analysis	Parameter	Healthy subjects (*N* = 24)					Noncall patients			Callosotomized patients				
		K–S test	Mean	SD	Prc 2.5	Prc 97.5	NP01	NP02	NP03	CP01	CP02	CP03	CP04	CP05
Slow-wave properties	Density	0.350	18.7	4.4	10.1	25.4	20.3	22.6	12.4	13.8	14.4	17.5	9.8	15.9
	Amplitude	0.410	50.3	15.6	32.3	96.0	62.9	35.6	42.8	55.6	78.6	***102.8***	69.4	50.3
	Slope	0.350	1.1	0.3	0.8	1.7	1.6	0.9	1.1	1.4	**1.8**	***2.5***	***2.2***	1.6
	Speed	0.991	2.3	0.3	1.8	2.9	2.1	2.2	2.1	1.9	2.5	1.9	**1.8**	***1.5***
*Involvement of PCA	Frontocentral	0.993	73.1	7.0	57.8	85.1	81.8	72.1	67.9	***32.6***	61.7	***50.4***	***47.0***	***26.8***
	Anterior/posterior	0.744	19.7	5.7	9.7	34.0	11.2	17.2	16.3	13.9	**8.5**	10.5	21.4	32.0
	Left/right	0.119	7.2	3.1	2.7	15.3	7.0	10.8	**15.8**	***53.6***	***29.8***	***39.1***	***31.5***	***41.2***
Traveling asymmetry	Cross-hemispheric propagation	0.830	63.2	3.5	54.9	69.0	65.8	57.3	57.8	***35.9***	***43.6***	***22.4***	***41.8***	***41.2***
	Asymmetry	0.652	36.8	2.4	43.3	33.4	35.8	**31.9**	33.4	***19.7***	***24.8***	***15.8***	***24.2***	***24.4***
*Asymmetry amplitude percentiles	Prc 0–20	0.414	34.6	2.4	31.1	41.2	31.3	**28.6**	**29.5**	***21.2***	***20.7***	***14.6***	***18.9***	***20.9***
	Prc 20–40	0.560	35.7	2.7	31.5	42.5	34.4	32.6	33.4	***20.7***	***23.8***	***15.0***	***24.0***	***23.6***
	Prc 40–60	0.622	36.5	2.7	32.3	43.7	36.5	**30.6**	33.1	***20.7***	***25.3***	***14.5***	***24.2***	***25.2***
	Prc 60–80	0.865	37.7	2.6	32.8	44.1	37.6	**32.2**	35.5	***19.7***	***27.4***	***16.6***	***27.5***	***25.4***
	Prc 80–100	0.696	39.8	2.1	35.6	45.5	39.0	35.7	36.0	***16.2***	***26.7***	***18.2***	***26.5***	***26.8***
Relative hemispheric difference	Density 75 µV	0.869	−0.3	0.7	−2.5	1.0	0.2	0.2	−0.5	**−*5.4***	**−*3.4***	−1.8	**−*4.6***	−1.5
	Density 40 µV	0.839	−0.3	0.8	−2.6	1.1	0.1	0.2	−1.0	**−*5.7***	**−*4.1***	−1.5	**−*5.7***	−1.6
	Origins	0.908	−0.5	0.7	−2.1	0.9	**1.0**	−0.2	***2.5***	**−*3.4***	**−*3.1***	−1.4	−0.3	**−*3.8***
Absolute hemispheric difference	Density 75 µV	0.382	1.7	0.5	1.1	2.9	1.9	**0.8**	2.0	***8.3***	***8.4***	***10.7***	***8.2***	***4.3***
	Density 40 µV	0.192	1.9	0.5	1.3	3.1	2.3	**1.1**	2.6	***8.2***	***9.5***	***11.3***	***9.7***	***4.9***
	Origins	0.967	4.4	0.6	3.0	5.5	4.1	5.0	***6.3***	***7.4***	***6.8***	***7.1***	3.7	5.5

Summary of statistics related to comparisons between patients and healthy subjects. The first two columns indicate the analyses of interest. Columns three to seven include descriptive statistics for the HS group: *p* value of the Kolmogorov–Smirnov (K–S) test for data normality, group-level mean (mean), SD of the mean (SD), 2.5 (Prc 2.5), and 97.5 (Prc 97.5) percentiles of the distribution. The columns for Noncall patients show the values of the parameter of interest observed in each of the three noncallosotomized patients (NP01–NP03). The columns for Callosotomized patients show the values of the parameter of interest observed in each of the five callosotomized patients (CP01–CP05). Bold text indicates values that fall off the 2.5–97.5 percentile range (α < 0.05). Bold and italic text indicates values that are significantly different from those of the HS group after Bonferroni correction. The correction was applied based on the number of tested subjects (*N* = 8). The exceptions are the analyses marked with *, for which the correction also took into account the number of related parameters that were tested. Prc, Percentile class.

### Data availability

Relevant data that support the findings of this study are available from the corresponding authors on motivated request.

## Results

### Sleep structure

**Figure 3.**
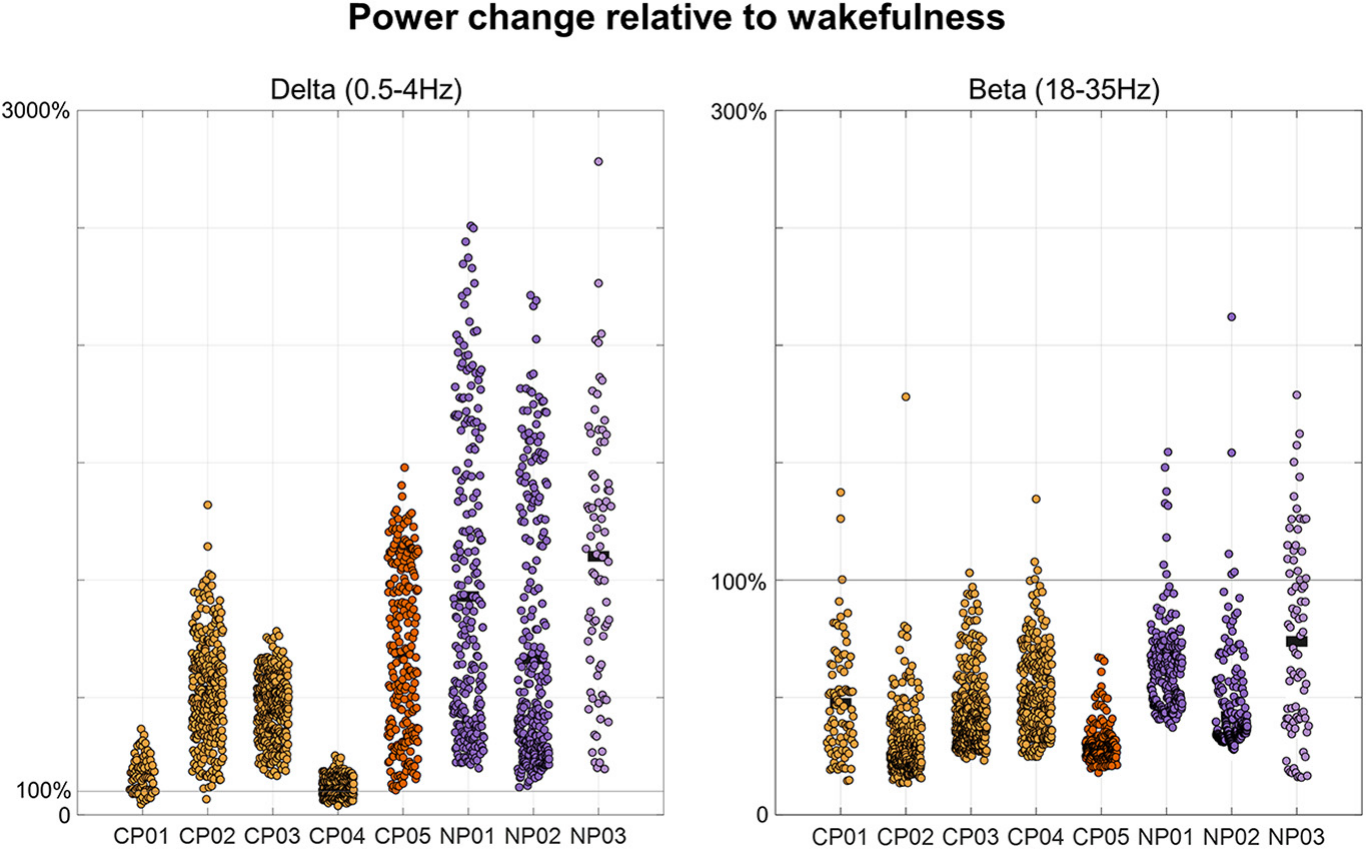
EEG power changes relative to wakefulness. The sleep stage classification in four of the five callosotomized patients (CP01–CP04) and of the NP with epilepsy (NP03) was made difficult by the presence of altered patterns of brain activity. However, the reliability of the sleep-scoring procedure is supported by the direct comparison of EEG power between epochs scored as NREM sleep and eyes-closed wake recordings collected before sleep, which showed an increase in SWA (0.5–4 Hz) and a decrease in high-frequency activity (beta, 18–35 Hz) in all CPs (CP01–CP05) and NPs (NP01–NP03) subjects. Here 100% corresponds to the signal power in wakefulness. The SWA increase was overall smaller in CPs relative to NPs. CPs are represented with orange dots (CP05 = dark orange), NPs with purple dots (NP03 = light purple).

Tables 1 and 2 report demographic and clinical characteristics for all patients. Table 1 also displays the sleep macrostructure in each patient and in the healthy control group. Of note, all epilepsy patients (CP01–CP05 and NP03) presented altered patterns of brain activity, with bursts of spike-wave discharges, during both wakefulness and sleep (Fig. 2). Such nonphysiological activity was particularly evident in four patients (CP01–CP04) and limited the possibility to accurately recognize changes in sleep depth based on standard criteria (the remaining patient, CP05, is marked using a distinctive color in figures). For this reason, a distinction between N2 and N3 sleep was not made. Examined NREM epochs in CPs were characterized by an increase in SWA (0.5–4 Hz) and a decrease in high-frequency activity (18–35 Hz) relative to wake epochs (Fig. 3), thus confirming the reliability of performed sleep scoring.

### Slow-wave characteristics

**Figure 4.**
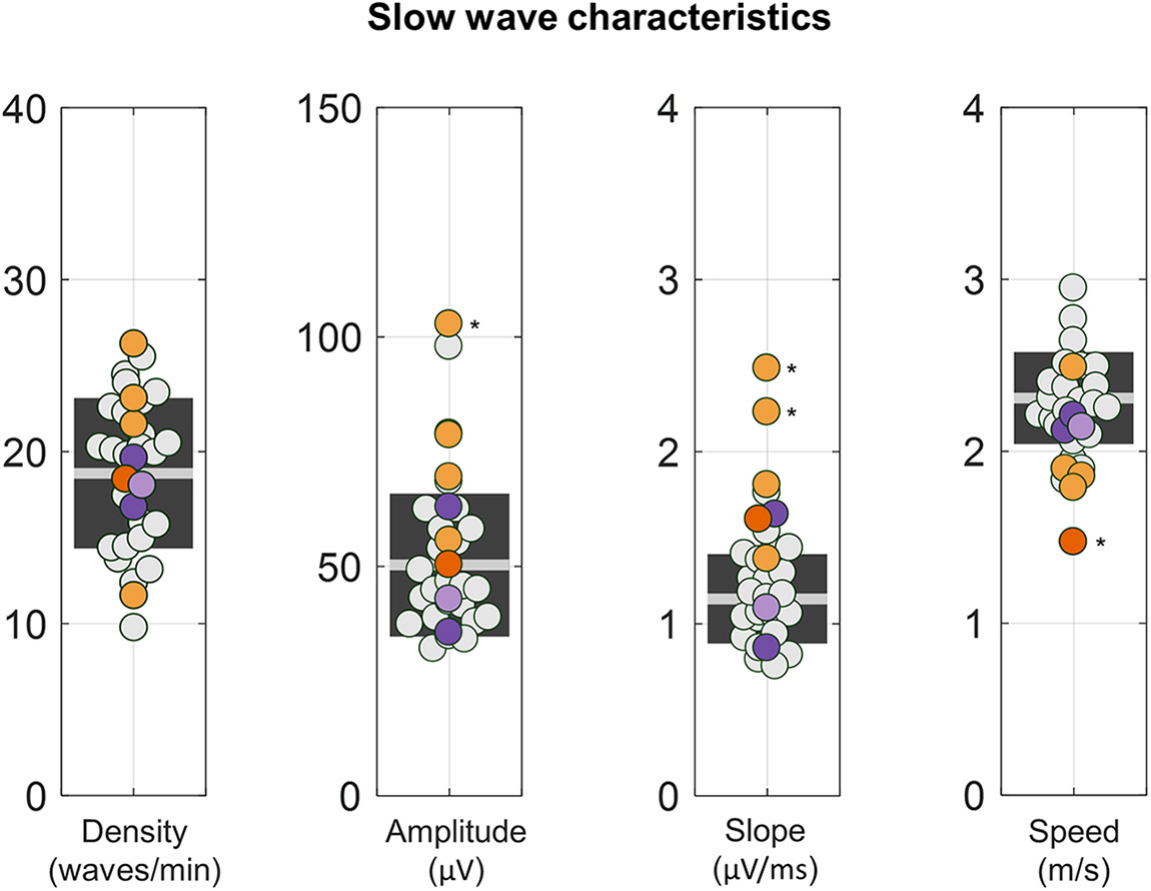
Properties of NREM slow waves. For each subject, we computed slow-wave density (in number of waves per minute), negative amplitude (in microvolts), descending slope (in microvolts per milliseconds), and propagation speed (in meters per second). CPs are represented with orange dots (CP05 = dark orange), NPs with purple dots (NP03 = light purple), and HSs with light gray dots. The light gray horizontal line represents the mean for the HSs, while the dark gray box represents 1 SD around the mean. Values observed in the eight patients (CPs and NPs) were compared with the 24 HSs. *p_corrected_ < 0.05.

Slow-wave density (CPs: 20.2 ± 5.6 waves/min; range, 11.7–26.3 waves/min; HSs: 18.7 ± 4.4 waves/min; range, 9.8–25.5 waves/min), amplitude (CPs: 62.3 ± 21.5 µV; range, 50.3–102.8 µV; HSs: 50.3 ± 15.6 µV; range, 32.1–97.9 µV), slope (CPs: 1.6 ± 0.5 µV/ms; range, 1.4–2.5 µV/ms; HSs: 1.1 ± 0.3 µV/ms; range, 0.8–1.8 µV/ms), and propagation speed (CPs: 2.0 ± 0.3 m/s, range 1.5–2.5 m/s; HSs: 2.3 ± 0.3 m/s; range, 1.8–2.9, m/s) did not differ between CPs and healthy control subjects as a group (Fig. 4). Specifically, we found no significant differences(*p*_corrected_ < 0.05) in slow-wave density (all *p*_uncorrected_ > 0.08, |*z*| < 1.7344), while significant effects were observed only inCP03 for amplitude (*p*_uncorrected_ = 0.008, |*z*| = 3.3688); in CP03 (*p*_uncorrected_ < 0.001, |*z*| = 5.1634) and CP04 (*p*_uncorrected_ < 0.001, |*z*| = 4.1840) for slope; and in CP05(*p*_uncorrected_ = 0.002, |*z*| = 3.1509) for speed. Results did not change after controlling for between-subject age differences, except for speed, in which no significant differences were found in any patients with respect to HSs (all *p*_uncorrected_ > 0.01, |*z*| < 2.5532).

### Slow-wave involvement

**Figure 5.**
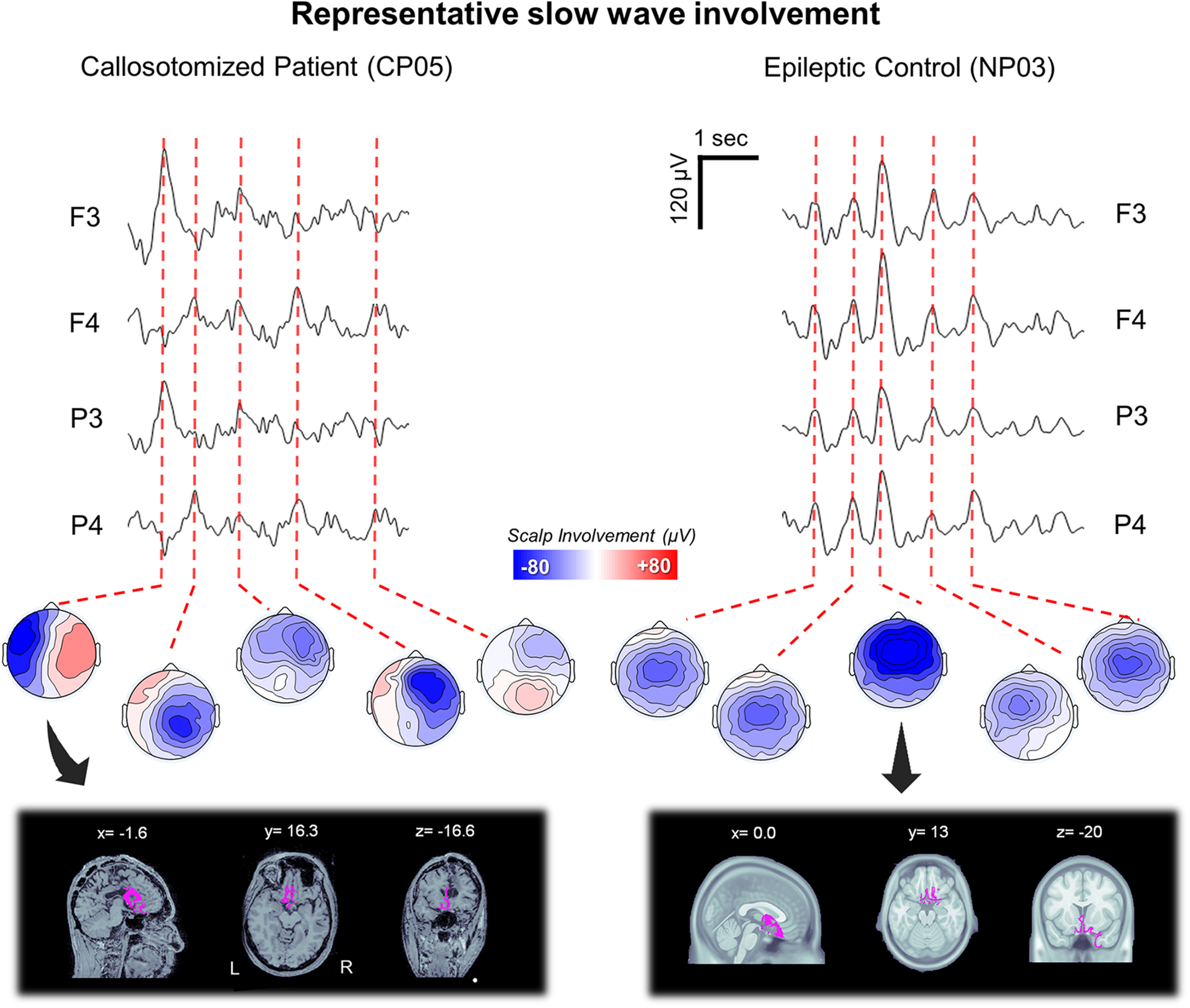
Representative slow-wave involvement patterns in a callosotomized (CP05) and a noncallosotomized (NP03) patient with epilepsy. Top, Representative NREM sleep EEG traces(5 s) for two left (F3, P3) and two right (F4, P4) channels and the relative scalp involvement associated with exemplar slow waves. Bottom, The source-reconstructed signal distributions for two representative slow waves. The source modeling has been performed using BrainStorm. A symmetric boundary element method (BEM) was used to define the forward model, while the inverse matrix was computed using the standardized low-resolution brain electromagnetic tomography (sLORETA) constraint. The cortical maps are thresholded at 80% of the maximum signal amplitude.

Visual inspection of EEG traces suggested that most sleep slow waves of CPs may present an asymmetric scalp distribution (Fig. 5).

**Figure 6.**
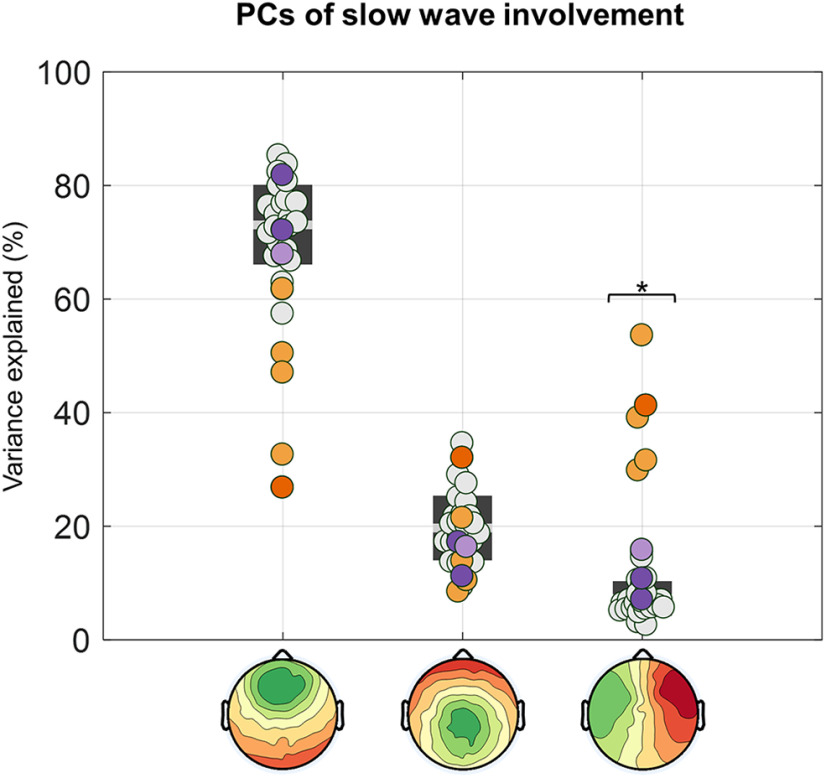
PCA-based analysis of slow-wave involvement. The involvement distribution (mean EEG signal calculated across all electrodes in a 40 ms window centered on the wave peak; in microvolts) of all slow waves was entered in a PCA. The plot shows the variance explained by each of the three PCs in all subjects. CPs are represented with orange dots (CP05 = dark orange), NPs with purple dots (NP03 = light purple), and HSs with light gray dots. *p_corrected_ < 0.05.

This observation was quantitatively confirmed through a PCA-based comparison of slow-wave involvement across groups (Fig. 6). In fact, in HSs the 95% of the variance related to scalp slow-wave involvement was explained by three PCs, with maxima located in the centrofrontal area (73.1 ± 7.0%; range, 57.4–85.3%, of the total variance explained by the first three components), anterior or posterior area (19.7 ± 5.7%; range, 9.4–34.6%), and the left or right hemisphere (7.2 ± 3.1%; range, 2.6–15.4%), respectively. Similar values were obtained in the NP group, with percentages corresponding to 73.9 ± 7.1% (range, 67.9–81.8%), 14.9 ± 3.3% (range, 11.2–17.2%), 11.2 ± 4.4% (range, 7.0–15.8%), respectively. On the other hand, in the CP group we observed a significant increase in the variance explained by the third (left/right) component (39.0 ± 9.5%; range, 29.8– 53.6%; *p*_corrected_ < 0.05, |*z*| > 7.3503; Bonferroni correction based on the number of tested subjects and PCs), at the expense of the other two symmetrical components (centrofrontal component: 43.7 ± 14.1%; range, 26.8–61.7%; anterior/posterior component: 17.2 ± 9.6%; range, 8.5–32.0%). In particular, the variance explained by the first component was significantly decreased in four (CP01, CP03, CP04, CP05) of five CPs.

### Cross-hemispheric propagation of slow waves

**Figure 7.**
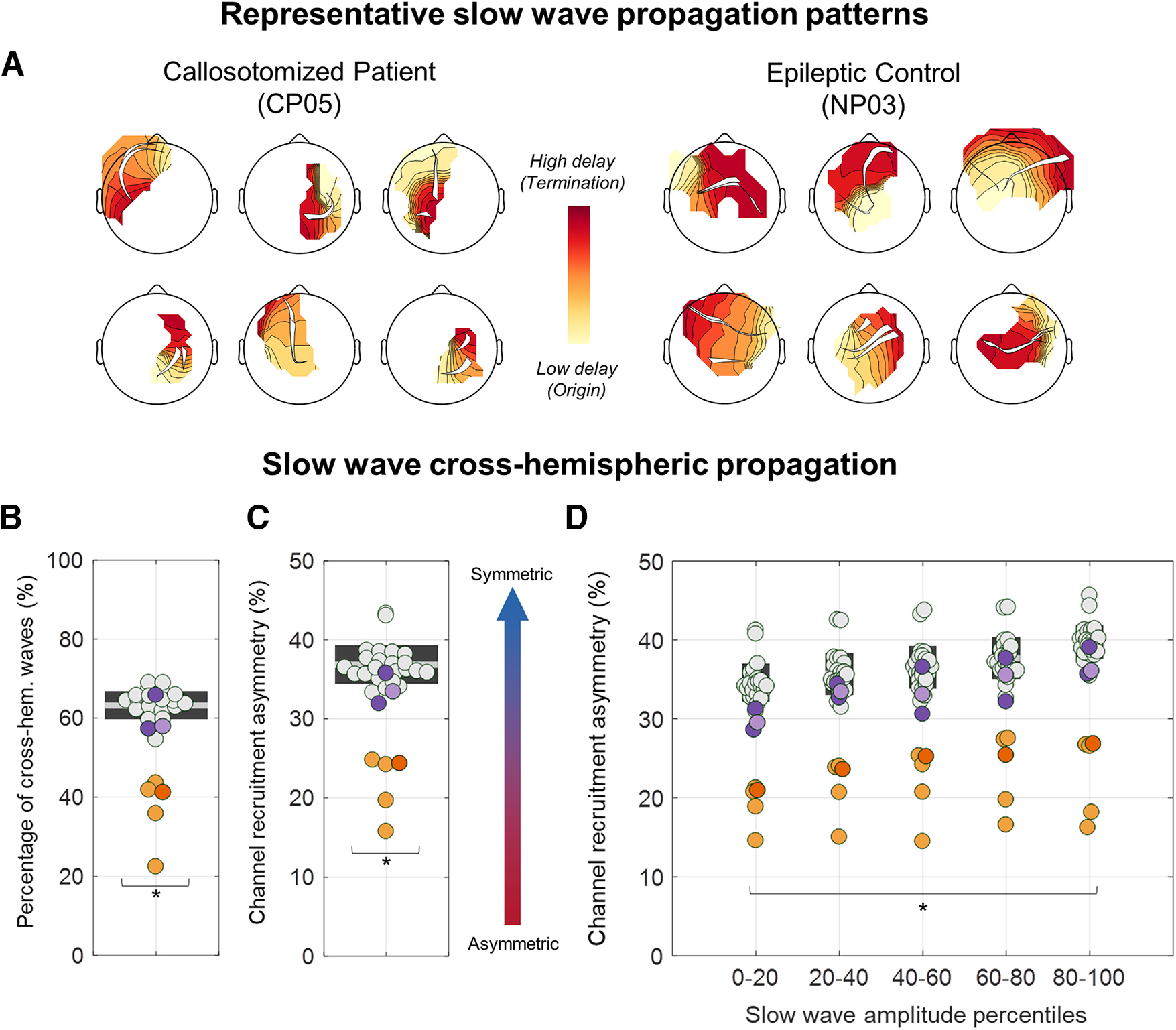
Quantitative analysis of slow-wave cross-hemispheric propagation. ***A***, Traveling delay maps and relative propagation streamlines of six representative slow waves of CP05 and NP03. In CPs, slow waves tended to remain confined to the origin hemisphere, while cross-hemispheric propagation was common in NPs and HSs. ***B***, The percentage of cross-hemispheric slow waves was computed as the number of slow waves for which at least one of the propagation streamlines passed the nasion–inion midline axis relative to the total number of detected slow waves. ***C***, The recruitment asymmetry was determined by computing the number of channels in the hemisphere with less recruited electrodes divided by the total number of recruited channels across the two hemispheres. Values close to 50% indicate a symmetric distribution, while values close to 0% indicate a unilateral wave. ***D***, This second parameter was also computed for slow waves grouped into five amplitude percentile classes (0–20, 20–40, 40–60, 60–80, and 80–100). CPs present a significantly reduced percentage of cross-hemispheric slow waves and an increased channel recruitment asymmetry (unihemispheric distribution). CPs are represented with orange dots (CP05 = dark orange), NPs with purple dots (NP03 = light purple), and HSs with light gray dots. *p_corrected_ < 0.05.

Next, we investigated whether alterations in the scalp distribution of slow waves in callosotomized patients could be explained by a lackof cross-hemispheric propagation of individual slow waves(Fig. 7*A*).

The percentage of slow waves presenting a cross-hemispheric propagation was significantly reduced in CPs (37.0 ± 8.6%; range, 22.4–43.6%) relative to HSs (63.2 ± 3.5%; range, 54.6–69.0%; *p*_corrected_ < 0.05, |*z*| > 5.5345; Fig. 7*B*). Consistent with this, slow waves in CPs showed a stronger lateralization in terms of the number of channels recruited along the propagation pattern in each of the two hemispheres (CPs: 21.8 ± 4.0%; range, 19.7–24.8%; HSs: 36.8 ± 2.4%; range, 33.4–43.3%; *p*_corrected_ < 0.05, |*z*| > 4.9546; Fig. 7*C*). Of note, such lateralization appeared to similarly affect all the slow waves regardless of their amplitude (*p*_corrected_ < 0.05; Bonferroni correction based on the number of tested subjects and amplitude percentile classes; Fig. 7*D*). Given that slow-wave traveling was computed by applying a spatiotemporal clusterization procedure that could have concealed potential propagation discontinuities caused by cortico-subcortico-cortical loops, the same analyses were repeated without this procedure. Obtained results confirmed the above observations by showing that all CPs had a lower proportion of cross-hemispheric slow waves (all *p*_uncorrected_ < 0.0001; |*z*| > 5.7083) and a stronger inter-hemispheric asymmetry in slow-wave spreading with respect to HSs (all *p*_uncorrected_ < 0.0001,|*z*| > 4.1063). All results remained significant after controlling for between-subjects age differences.

**Figure 8.**
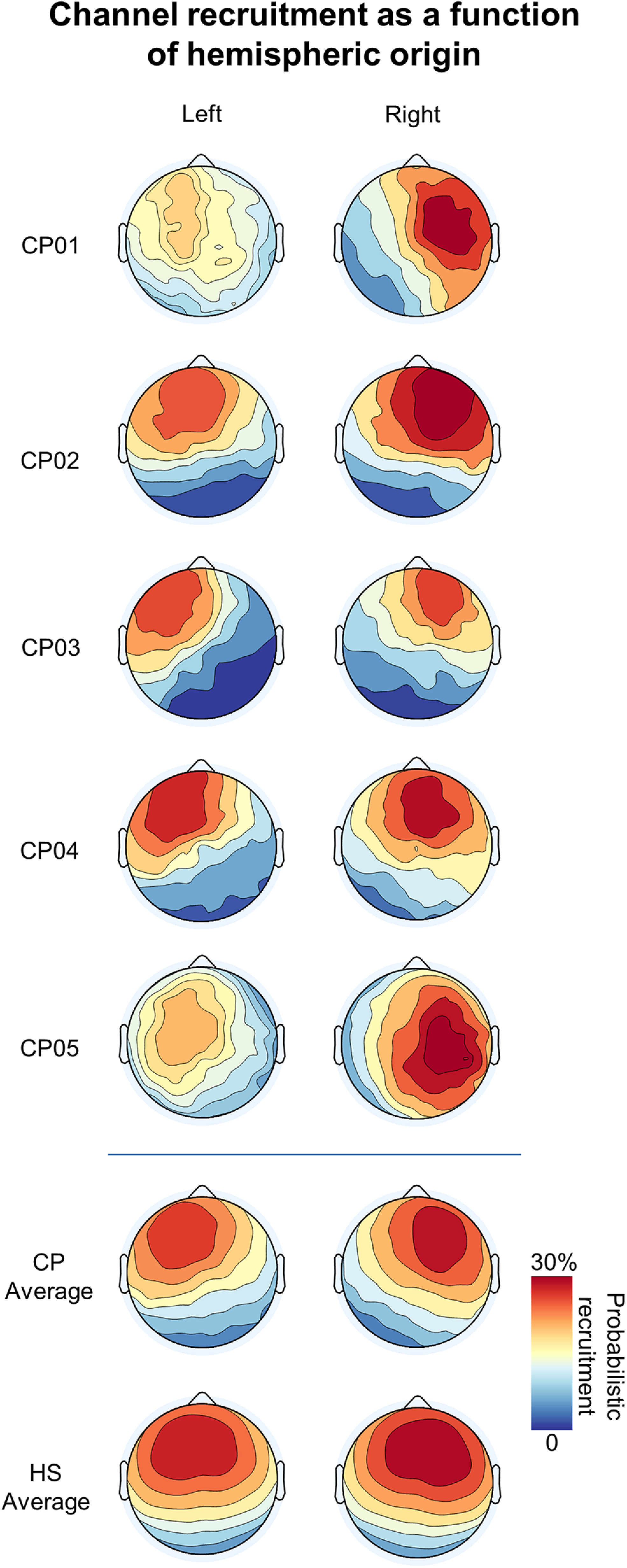
Scalp probabilistic recruitment as a function of hemispheric origin. Top, For each of the CPs, the probabilistic recruitment (probability of channel recruitment in a slow wave) for slow waves originating in the left (left column) or right (right column) hemisphere. The probabilistic recruitment is expressed as a percentage with respect to the total number of detected slow waves, regardless of their origin site. Bottom, The average probabilistic recruitment for CPs and HSs. It is evident that the involvement tended to be more symmetrical in HSs with respect to CPs. Moreover, this analysis suggested a relatively stronger recruitment of the right (vs left) hemisphere in both CPs and HSs.

Figure 8 shows the probabilistic channel recruitment of slow waves originating in the left or right hemisphere in each of the CP individuals and in the HS group. This qualitative representation further shows that slow waves tended to remain confined to the origin hemisphere in callosotomized but not in control subjects.

### Inter-hemispheric differences in slow-wave latency

**Figure 9.**
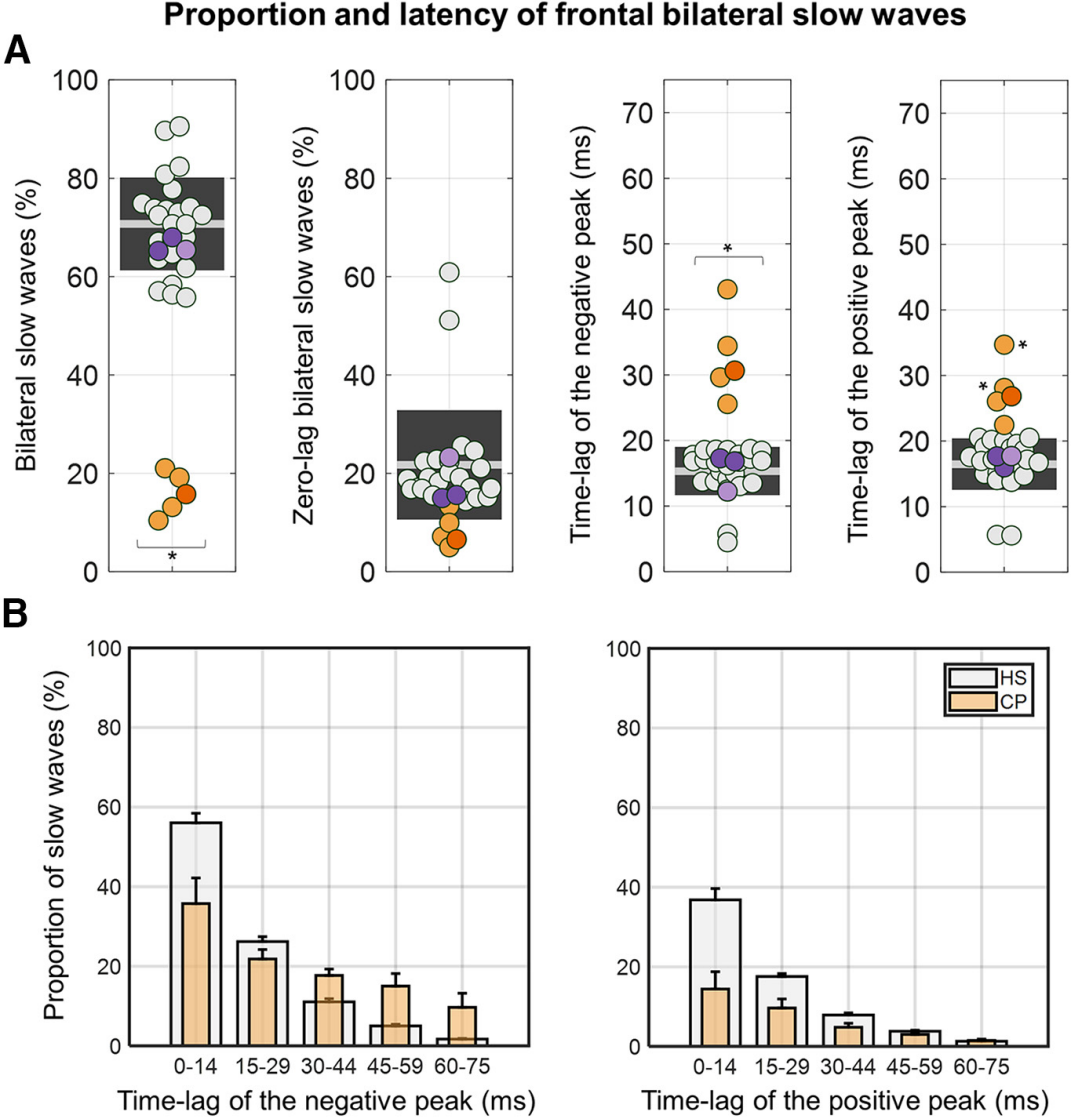
Slow-wave synchronization across symmetrical frontal electrodes. ***A***, The four plots respectively show (from left to right) the percentage of bilaterally detected slow waves, the proportion of perfectly synchronous (zero-lag) slow waves that may reflect volume conduction, the absolute time-lag between the negative peaks of bilateral slow waves, and the absolute time-lag between the positive peaks of bilateral slow waves. ***B***, Distribution of time-lags for negative (left) and positive (right) peaks in HSs and CPs. Note that the percentages were calculated with respect to the total number of bilateral negative peaks and that bilateral positive peaks were not found in the explored time window for some of these slow waves. *p_corrected_ < 0.05.

Given that cross-hemispheric propagation was reduced but not abolished in CPs, we investigated whether this depends on an apparent synchronization caused by volume conduction of EEG signals, or on a real spreading of slow waves through alternative pathways. To this aim, we analyzed the co-occurrence and degree of synchronization of slow waves detected in homologous frontal electrodes (Fig. 9). In line with results described above, we found that the percentage of bilateral slow waves was significantly reduced in CPs with respect to HSs (*p*_corrected_ < 0.05, all *p*_uncorrected_ < 0.0001, |*z*| > 5.2439; Fig. 9*A*). Moreover, the time-lag between negative peaks of bilateral detections was significantly higher in CPs relative to HSs(*p*_uncorrected_ < 0.0055, |*z*| > 2.7790; Fig. 9*A*), and these results remained significant after adjustment for inter-subject age differences. A similar trend toward an increased time-lag in CPs was found for positive peaks, although the difference with respect to HSs reached significance only in CP01 (*p*_uncorrected_ = 0.0030, |*z*| = 2.9679) and CP03 (*p*_uncorrected_ < 0.0001, |*z*| = 4.6526; Fig. 9*A*). After adjustment for age differences a significant effect was found also in CP02 (*p*_uncorrected_ = 0.0013, |*z*| = 3.2269). Finally, we found no significant differences between CPs and HSs with respect to the proportion of slow waves showing perfectly synchronous (zero-lag) negative peaks across the two hemispheres (*p*_uncorrected_ > 0.1322, |*z*| < 1.5055; Fig. 9*B*). Of note, for this latter analysis the distribution of HSs included a clear outlier (HS10; value >3 SDs from the group mean), but results did not change after exclusion of this subject.

### Inter-hemispheric asymmetry in sleep depth

**Figure 10.**
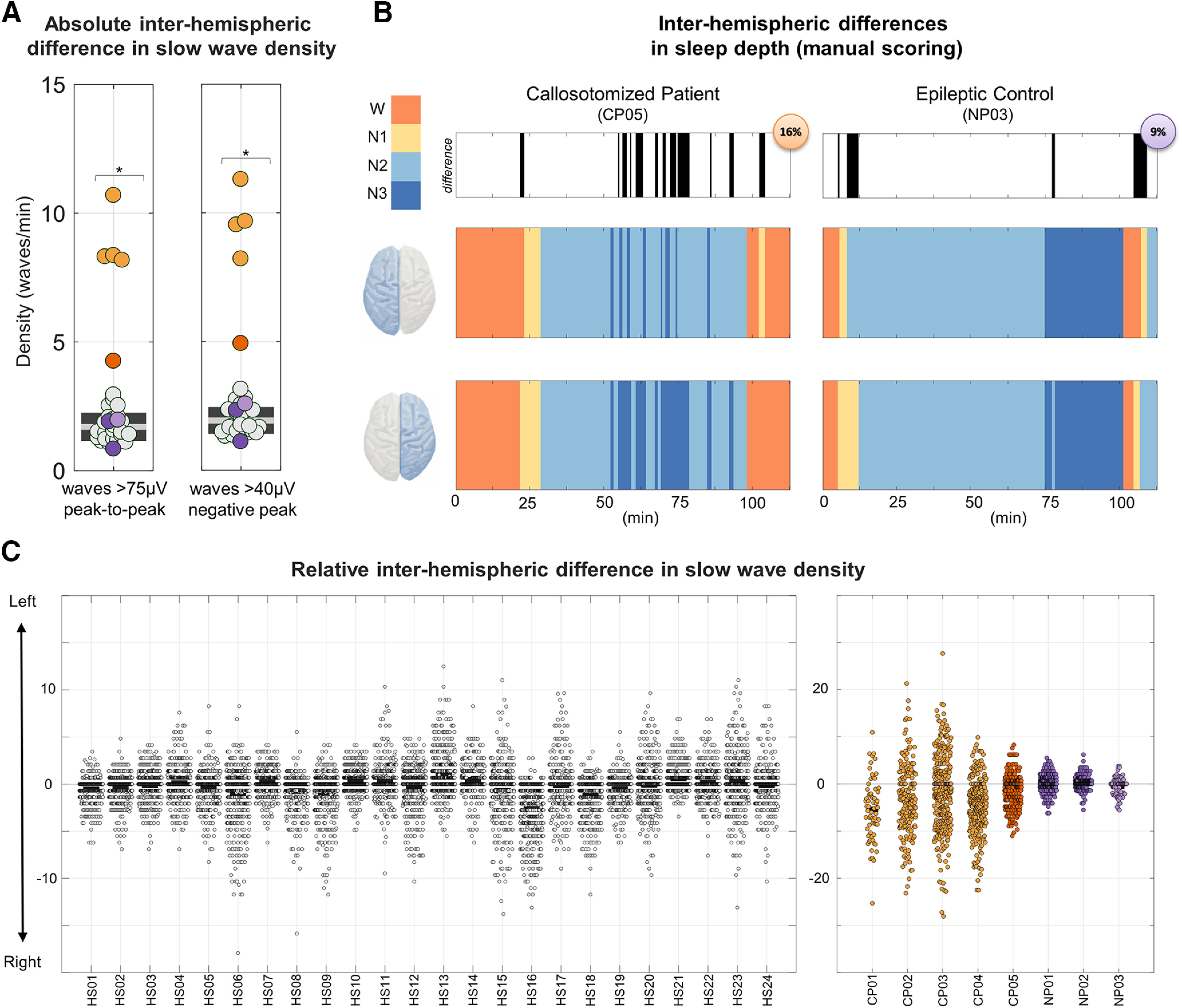
Inter-hemispheric difference in slow-wave density. ***A***, Absolute inter-hemispheric difference in slow-wave density. The plot on the left shows the absolute inter-hemispheric (|left-right|) difference in slow-wave density. CPs are represented with orange dots (CP05 = dark orange), NPs with purple dots (NP03 = light purple), and HSs with light gray dots. *p_corrected_ < 0.05. ***B***, Sleep scoring (120 min; 0 = time of lights off) in a callosotomized patient (CP05) and in the noncallosotomized patient with epilepsy (NP03). Sleep scoring was performed separately for each hemisphere (i.e., using only electrodes of the left or right side) by an operator blind to both the identity of the subjects and the evaluated brain hemisphere. In the top panel, black sections indicate epochs for which different stages were scored across the two hemispheres. Bottom panels represent the sleep scoring for the first sleep cycle in the two patients and for each hemisphere (left top, right bottom). ***C***, Difference in the mean slow-wave density (in waves per minute) across three left (F3, C3, P3) and three right (F4, C4, P4) channels. A peak-to-peak amplitude threshold corresponding to 75 µV was applied to minimize spurious cross-hemispheric detection caused by simple volume conduction. Each dot represents a different NREM sleep epoch. The left plot represents each of the HSs (HS01–HS24), while the right plot shows the CPs (CP01–CP05) and the NPs (NP01–NP03). Lower (negative) values indicate a higher number of slow waves detected in the right hemisphere. In both the CP and HS groups there was a tendency toward a higher slow-wave density in the right relative to the left hemisphere.

Last, we investigated whether the lack of strong inter-hemispheric connections could be responsible for an unbalanced sleep depth—as reflected by the generation and synchronization of sleep slow waves—across the two hemispheres. To this aim, we first tested whether CPs and control HSs presented an asymmetric incidence of large-amplitude slow waves, characterized by peak-to-peak (negative-to-positive) amplitude of >75 µV. Specifically, we computed for each sleep epoch the relative difference in slow-wave incidence across homologous electrodes of the two hemispheres. We found that, in both groups, many of the NREM sleep epochs were characterized by an inter-hemispheric difference in slow-wave density (Fig. 10*A*,*C*), with a relative hemispheric dominance that varied from epoch to epoch. Overall, however, slow-wave density tended to be higher in the right, relative to the left, hemisphere in both CPs (−3.38 ± 0.69 waves/min, difference left–right) and HSs (−0.26 ± 0.15 waves/min), although the effect reached statistical significance only in the first group (one-sample *t* tests against the null hypothesis of no asymmetry; HSs: *p* = 0.099, |*t*_(23)_| = 1.7197, bCIs = −0.55, 0.01; CPs: *p* = 0.012, |*t*_(4)_| = 4.3676, bCIs = −4.84, −2.08). Similar results were obtained using a slow-wave amplitude threshold corresponding to a negative amplitude of 40 µV (HSs: *p* < 0.074,|*t*_(23)_| = 1.8735, bCIs = −0.65, −0.01; CPs: *p* = 0.017, |*t*_(4)_| = 3.9427, bCIs = −5.39, −2.04). Importantly, the relative inter-hemispheric asymmetry was not systematically different across CPs and HSs (Table 3). However, when the absolute (|left – right|), rather than the relative (left – right) inter-hemispheric difference in slow-wave incidence was considered, this parameter was significantly greater in CPs (5.6 ± 3.8 waves/min) relative to HSs (1.7 ± 0.5 waves/min, *p*_corrected_ < 0.05, |*z*| > 4.6552; Fig. 10*A*). Similar results were obtained for the 40 µV amplitude threshold (HSs = 1.9 ± 0.5 waves/min; CPs = 6.2 ± 3.9 waves/min; *p*_corrected_ < 0.05, |*z*| > 5.8069). All results remained significant after controlling for between-subjects age differences.

**Figure 11.**
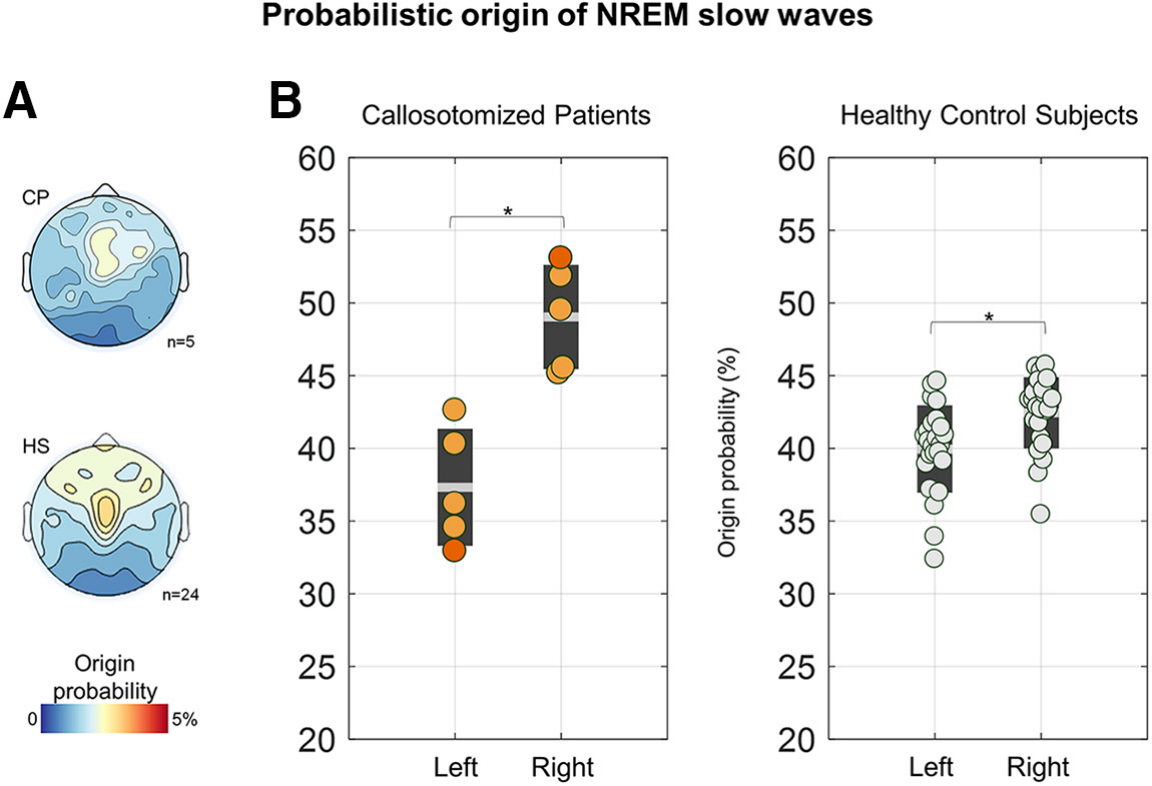
Differences in probabilistic origin across the left and right hemispheres. ***A***, Topographic map of probabilistic slow-wave origin in CPs (top) and HSs (bottom). Similar distributions, with maxima in central–lateral and anterior areas were found in both CPs and HSs. ***B***, A higher proportion of slow waves originated in the right versus left hemisphere in both CPs and HSs. CPs are represented with orange dots (CP05 = dark orange), NPs with purple dots (NP03 = light purple), and HSs with light gray dots. *p_corrected_ < 0.05.

In light of the above observations, we then asked whether the greater inter-hemispheric differences in slow-wave incidence found in CPs could be better explained by a more disproportionate slow-wave generation across brain hemispheres, or simply by the lack of cross-hemispheric propagation. To this aim, we first determined the overall proportion of slow waves with a clear origin in the left or in the right hemisphere with respect to the total number of detected slow waves (Fig. 11). Consistent with the above-reported results, we found that a greater percentage of waves originated in the right hemisphere in both HSs (paired *t* test, *p* = 0.009, |*t*_(23)_| = 2.8471, bCIs = −4.11, −0.85; left = 39.95 ± 2.99%, right = 42.45 ± 2.47%) and CPs (paired *t* test, *p* = 0.03, |*t*_(4)_| = 3.4595, bCIs = −17.64, −5.58; left = 37.33 ± 4.04%, right = 49.03 ± 3.59%). However, the relative and the absolute inter-hemispheric asymmetry in origin density did not show systematic differences across the two groups (Table 3). In fact, for both parameters, only three of the five CPs displayed a statistically significant difference with respect to HSs. Moreover, a significant difference was also found in one NP, thus indicating that differences found in the three CPs were not specific.

## Discussion

The slow waves of NREM sleep have been shown to spread across brain areas in scalp hd-EEG recordings of healthy human individuals (Massimini et al., 2004; Murphy et al., 2009). While this macroscale cortical traveling has been thought to be mediated by cortico-cortical white matter connections, to date only indirect correlational evidence has supported this assumption (Buchmann et al., 2011; Piantoni et al., 2013). Furthermore, findings reported in the literature are contradictory, likely because of methodological discrepancies and limitations. In the present study, we show that a complete resection of the CC, which contains the main bundle of inter-hemispheric white matter fibers, is associated with an increased incidence of unihemispheric slow waves, reflecting a decrease in cross-hemispheric propagation. Interestingly, our results also demonstrate that slow waves originate more often in the right relative to the left hemisphere and that this asymmetry is not significantly affected by the resection of the CC.

### The corpus callosum is essential for the cross-hemispheric propagation of sleep slow waves

Here we demonstrate that the cross-hemispheric propagation of NREM slow waves largely depends on the integrity of callosal white matter tracts. Indeed, while in healthy adult subjects >60% of all slow waves showed a clear cross-hemispheric propagation, in callosotomized patients >60% of them remained confined within the cerebral hemisphere in which they originated. These results are in line with previous correlational evidence, indicating a direct relationship between parameters reflecting slow-wave synchronization and the micro-structure of the anterior CC (Buchmann et al., 2011; Piantoni et al., 2013; but see Sanchez et al., 2019). More in general, they provide support to the hypothesized relationship between patterns of slow-wave propagation and structural cortico-cortical connectivity (Murphy et al., 2009; Kurth et al., 2017; Schoch et al., 2018). In this respect, our findings may appear in contrast with recent work showing a positive correlation between indices reflecting white matter damage and slow-wave synchronization efficiency in TBI patients (Sanchez et al., 2019). These discrepancies may in part be explained by the different methodological approach, as the indices investigated in the previous work (slow-wave amplitude/slope) only indirectly reflect slow-wave synchronization/propagation across brain areas. Another possibility is that the impact of white matter integrity may change as a function of lesion extension and involved pathways. Indeed, a damage-related “disconnection” in TBI patients may enhance the cortical propensity to locally generate and synchronize slow-wave-like events, as shown in animal models of cortical deafferentation (Topolnik et al., 2003; Timofeev et al., 2000, 2013). In fact, the neuronal bistable state typically observed during sleep has been suggested to represent a “default” state of isolated neocortical modules (Sanchez-Vives and McCormick, 2000; Lemieux et al., 2014; Capone et al., 2019), although the thalamus and other subcortical structures seem to provide a significant contribution to shaping and synchronizing slow cortical oscillations in physiological sleep (Neske, 2016; Gent et al., 2018; Vantomme et al., 2019). In this light, extensive white matter lesions, such as those observed in TBI patients, may favor the transition of many cortical neurons into a bistable state, thus determining a paradoxical increase in slow-wave generation and local synchronization. The slow-wave cortical traveling has been suggested to have a direct role in organizing information processing and plasticity in cortical networks through the local modulation of spindles and high-frequency activity (Cox et al., 2014). Thus, the alteration of cross-hemispheric propagation may lead to an alteration of plasticity-related processes requiring the interaction and coordination of activity across the two brain hemispheres. It should be noted, however, that the resection of the CC did not completely abolish cross-hemispheric slow wave propagation. While this residual bilateral cortical involvement may in part represent a spurious consequence of volume conduction, our results suggest that this issue alone is not sufficient to explain the full magnitude of the phenomenon. In fact, the proportion of perfectly synchronous (zero-lag) bilateral slow waves was not significantly affected by callosotomy. Instead, we observed a longer inter-hemispheric delay between slow-wave negative peaks in callosotomized patients, which is consistent with the involvement of polysynaptic propagation pathways possibly including cortico-subcortico-cortical loops (Timofeev and Steriade, 1996). Additional mechanisms underlying residual bilateral slow waves in callosotomized patients may include direct subcortico-cortical recruitment processes (Siclari et al., 2014; Bernardi et al., 2018) and/or the involvement of anterior and posterior commissures (Mancuso et al., 2019), which were relatively spared in all the CP individuals. Interestingly, a similar change in inter-hemispheric delay was not observed for the positive peak occurring at the transition into the up-state. This observation is consistent with a role of subcortical structures in synchronizing up-state transitions across cortical neuronal populations (Lemieux et al., 2014; Neske, 2016).

### The resection of the corpus callosum is not sufficient for the manifestation of unihemispheric sleep

Our study showed that, during NREM sleep, large slow waves are often asymmetrically distributed across the two hemispheres in healthy adult individuals. Interestingly, the absolute degree of inter-hemispheric asymmetry is significantly increased in callosotomized patients. Based on the standard definition of sleep stages, this particular condition could lead to apparent differences in sleep depth across the two brain hemispheres. Such an asymmetry could be explained either by a change in the number of slow waves originated in the two hemispheres, or simply by the loss of cross-hemispheric propagation after callosotomy. However, since the cortical distribution of slow-wave origins was not systematically and significantly affected by the resection of the CC, the observed asymmetry could be entirely explained by the reduced cross-hemispheric slow-wave propagation. This observation is in line with previous evidence suggesting that the lack or resection of inter-hemispheric connections is not sufficient for the manifestation of unihemispheric sleep (Berlucchi, 1966; Montplaisir et al., 1990; Nielsen et al., 1993), as naturally seen in some animal species, such as birds and cetaceans (Rattenborg et al., 2000; Mascetti, 2016). On the other hand, the absence (as in birds) or small size (as in cetaceans; Tarpley and Ridgway, 1994) of the CC may prevent the cross-hemispheric spreading of sleep slow waves, and thus represent one fundamental prerequisite for unihemispheric sleep.

### Slow waves originate more often in the right than in the left hemisphere

Present results revealed that during NREM sleep, slow waves tend to originate more often in the right than in the left hemisphere in healthy adult subjects as well as in callosotomized patients, although the relative hemispheric predominance also varies from epoch to epoch. A similar inter-hemispheric difference in SWA during NREM sleep has been reported in some previous investigations (Goldstein et al., 1972; Sekimoto et al., 2000, 2007). Interestingly, our observation of a similar lateralization in patients who underwent callosal resection implies that such slow-wave lateralization does not depend on competitive regulatory mechanisms acting across the two hemispheres. Why then does the right hemisphere generate more slow waves than the left one during NREM sleep? In light of the homeostatic mechanisms that regulate SWA (Borb and Achermann, 1999) and of the known differences in hemispheric functional specialization (Karolis et al., 2019), the right hemisphere may develop a stronger function- and use-dependent “sleep need” during wakefulness that translates into higher slow-wave activity during subsequent sleep. However, this possibility is at odds with previous findings indicating a stronger rebound in SWA within the left hemisphere following extended wakefulness, relative to baseline sleep conditions (Achermann et al., 2001; Ferrara et al., 2002; Vyazovskiy et al., 2002). Of note, recent work showed that the first night of sleep in a new environment may be associated with an increased sleep-depth asymmetry, with the left hemisphere operating as a “night watch” (Tamaki et al., 2016). This observation raises the interesting possibility of a constitutional difference in the arousal-related, bottom-up control of sleep in the two hemispheres. One could speculate that a “deeper sleep” of the right hemisphere, which is highly involved in attentional control, may enable a relative disengagement from environmental stimuli (Bareham et al., 2014), while a “more awake” left hemisphere could facilitate the recognition of potentially relevant communicative stimuli that are especially important in social animals (Legendre et al., 2019). More specific studies will be required to directly test these hypotheses.

### Limitations

The main limitation of this study is the relatively small sample size. However, it should be emphasized that patients who underwent complete callosotomy represent an exceptionally rare population (Fabriet al., 2017). Furthermore, to overcome potential limitations related to the sample size, we performed evaluations at the single-subject level and applied strict criteria for the definition of “significant” group differences. Another potential limitation is that all the epilepsy patients (CP01–CP05 and NP03) presented alterations in the background EEG activity caused by the underlying pathologic condition. All ofthe signals have been carefully inspected to discard segments containing nonphysiological activity. Though it is still possible for some slow-wave-like epileptic events to have been included in our analyses. In addition, the use of medications, including antiepileptic and hypnotic drugs (Table 2), may also have affected recorded EEG signals. Although we cannot completely exclude the influence of these factors on our analyses, all results were consistent across a heterogeneous sample of callosotomized patients with distinct underlying conditions, comorbidities,and pharmacological therapies. Moreover, the noncallosotomized patients, including a subject with epilepsy, who were studied under similar conditions did not show the same pattern of slow-wave differences observed between callosotomized patients and the healthy adult control group. Finally, given that all patients in our sample and most of the healthy control subjects were right-handed, an investigation of the role of handedness in modulating slow-wave propagation or lateralization was not possible. Future studies will be necessary to shed light on this issue.

### Conclusions

This study systematically investigated the origin, distribution, and traveling of sleep slow waves in complete split-brain patients. To the best of our knowledge, our results are the first demonstration that the resection of inter-hemispheric connections significantly limits the cross-hemispheric propagation of sleep slow waves without affecting the relative distribution of slow-wave origins across the two hemispheres. These findings also provide further support to previous assumptions regarding the dependence of slow waves on cortico-cortical connections for their macroscale spreading. In light of previous evidence indicating that slow waves may modulate, throughout their propagation, spindle and high-frequency activity potentially related to plastic processes, our results indicate that callosotomy may significantly affect these sleep-dependent mechanisms. On a different perspective, our findings also demonstrate that the loss of inter-hemispheric connections in adult life is not sufficient, per se, to allow the appearance of unihemispheric sleep in humans, thus implying that in animals showing this particular behavioral state additional functional and/or anatomic mechanisms may play a pivotal role.

## References

[B1] Achermann P, Borbély AA (2003) Mathematical models of sleep regulation. Front Biosci 8:s683–s693. 10.2741/1064 12700054

[B2] Achermann P, Finelli LA, Borbély AA (2001) Unihemispheric enhancement of delta power in human frontal sleep EEG by prolonged wakefulness. Brain Res 913:220–223. 10.1016/s0006-8993(01)02796-2 11549390

[B3] Bareham CA, Manly T, Pustovaya OV, Scott SK, Bekinschtein TA (2014) Losing the left side of the world: rightward shift in human spatial attention with sleep onset. Sci Rep 4:5092. 10.1038/srep05092 24867667 PMC4035582

[B4] Berlucchi G (1966) Electroencephalographic studies in “split brain” cats. Electroencephalogr Clin Neurophysiol 20:348–356. 10.1016/0013-4694(66)90003-4 4143672

[B5] Bernardi G, Siclari F, Handjaras G, Riedner BA, Tononi G (2018) Local and widespread slow waves in stable NREM sleep: evidence for distinct regulation mechanisms. Front Hum Neurosci 12:1–13. 29970995 10.3389/fnhum.2018.00248 PMC6018150

[B6] Bernardi G, Betta M, Cataldi J, Leo A, Haba-Rubio J, Heinzer R, Cirelli C, Tononi G, Pietrini P, Ricciardi E, Siclari F (2019a) Visual imagery and visual perception induce similar changes in occipital slow waves of sleep. J Neurophysiol 121:2140–2152. 10.1152/jn.00085.2019 30943100

[B7] Bernardi G, Betta M, Ricciardi E, Pietrini P, Tononi G, Siclari F (2019b) Regional delta waves in human rapid-eye movement sleep. J Neurosci 39:2686–2697. 10.1523/JNEUROSCI.2298-18.2019 30737310 PMC6445986

[B8] Borb AA, Achermann P (1999) Sleep homeostasis and models of sleep regulation. J Biol Rhythms 14:559–570. 10.1177/074873099129000894 10643753

[B9] Buchmann A, Kurth S, Ringli M, Geiger A, Jenni OG, Huber R (2011) Anatomical markers of sleep slow wave activity derived from structural magnetic resonance images. J Sleep Res 20:506–513. 10.1111/j.1365-2869.2011.00916.x 21435064

[B10] Capone C, Rebollo B, Muñoz A, Illa X, Giudice P, Del Sanchez-Vives MV, Mattia M (2019) Slow waves in cortical slices: how spontaneous activity is shaped by laminar structure. Cereb Cortex 29:319–335. 10.1093/cercor/bhx326 29190336

[B11] Corsi-Cabrera M, Ondarza R, Martínez-Gutiérrez V, Río-Portilla Y, del Guevara MA, Ramos-Loyo J (2006) Role of corpus callosum in interhemispheric coherent activity during sleep. Clin Neurophysiol 117:1826–1835. 10.1016/j.clinph.2006.05.008 16807092

[B12] Cox R, Van Driel J, De Boer M, Talamini LM (2014) Slow oscillations during sleep coordinate interregional communication in cortical networks. J Neurosci 34:16890–16901. 10.1523/JNEUROSCI.1953-14.2014 25505340 PMC6608509

[B13] Delorme A, Makeig S (2004) EEGLAB: an open source toolbox for analysis of single-trial EEG dynamics including independent component analysis. J Neurosci Methods 134:9–21. 10.1016/j.jneumeth.2003.10.009 15102499

[B14] Fabri M, Foschi N, Pierpaoli C, Polonara G (2017) Split-brain human subjects BT. In: Lateralized brain functions: methods in human and non-human species (Rogers LJ, Vallortigara G, eds), pp 29–78. New York: Springer.

[B15] Ferrara M, De Gennaro L, Curcio G, Cristiani R, Bertini M (2002) Interhemispheric asymmetry of human sleep EEG in response to selective slow-wave sleep deprivation. Behav Neurosci 116:976–981. 10.1037//0735-7044.116.6.976 12492296

[B16] Gent TC, Bandarabadi M, Herrera CG, Adamantidis AR (2018) Thalamic dual control of sleep and wakefulness. Nat Neurosci 21:974–984. 10.1038/s41593-018-0164-7 29892048 PMC6438460

[B17] Goldstein L, Stoltzfus NW, Gardocki JF (1972) Changes in interhemispheric amplitude relationships in the EEG during sleep. Physiol Behav 8:811–815. 10.1016/0031-9384(72)90289-2 4339962

[B18] Hablitz LM, Vinitsky HS, Sun Q, Stæger FF, Sigurdsson B, Mortensen KN, Lilius TO, Nedergaard M (2019) Increased glymphatic influx is correlated with high EEG delta power and low heart rate in mice under anesthesia. Sci Adv 5:eaav5447. 10.1126/sciadv.aav5447 30820460 PMC6392807

[B19] Haxby JV, Guntupalli JS, Connolly AC, Halchenko YO, Conroy BR, Gobbini MI, Hanke M, Ramadge PJ (2011) A common, high-dimensional model of the representational space in human ventral temporal cortex. Neuron 72:404–416. 10.1016/j.neuron.2011.08.026 22017997 PMC3201764

[B20] Iber C, Ancoli-Israel S, Chesson AL, Quan S (2007) The AASM manual for the scoring of sleep and associated events: rules, terminology and technical specifications. Darien, IL: American Academy of Sleep Medicine.

[B21] Karolis VR, Corbetta M, Thiebaut de Schotten M (2019) The architecture of functional lateralisation and its relationship to callosal connectivity in the human brain. Nat Commun 10:1417. 10.1038/s41467-019-09344-1 30926845 PMC6441088

[B22] Kayser J, Tenke CE (2006) Principal components analysis of Laplacian waveforms as a generic method for identifying ERP generator patterns: I. Evaluation with auditory oddball tasks. Clin Neurophysiol 117:348–368. 10.1016/j.clinph.2005.08.034 16356767

[B23] Kuks JBM, Vos JE, O'Brien MJ (1987) Coherence patterns of the infant sleep EEG in absence of the corpus callosum. Electroencephalogr Clin Neurophysiol 66:8–14. 10.1016/0013-4694(87)90132-5 2431870

[B24] Kurth S, Riedner BA, Dean DC, O'Muircheartaigh J, Huber R, Jenni OG, Deoni SCL, LeBourgeois MK (2017) Traveling slow oscillations during sleep: a marker of brain connectivity in childhood. Sleep 40:zsx121 10.1093/sleep/zsx121 PMC5806587 28934529

[B25] Legendre G, Andrillon T, Koroma M, Kouider S (2019) Sleepers track informative speech in a multitalker environment. Nat Hum Behav 3:274–283. 10.1038/s41562-018-0502-5 30953006

[B26] Lemieux M, Chen JY, Lonjers P, Bazhenov M, Timofeev I (2014) The impact of cortical deafferentation on the neocortical slow oscillation. J Neurosci 34:5689–5703. 10.1523/JNEUROSCI.1156-13.2014 24741059 PMC3988418

[B27] Mancuso L, Uddin LQ, Nani A, Costa T, Cauda F (2019) Brain functional connectivity in individuals with callosotomy and agenesis of the corpus callosum: a systematic review. Neurosci Biobehav Rev 105:231–248. 10.1016/j.neubiorev.2019.07.004 31412269

[B28] Mascetti GG (2016) Unihemispheric sleep and asymmetrical sleep: behavioral, neurophysiological, and functional perspectives. Nat Sci Sleep 8:221–238. 10.2147/NSS.S71970 27471418 PMC4948738

[B29] Mascetti L, Muto V, Matarazzo L, Foret A, Ziegler E, Albouy G, Sterpenich V, Schmidt C, Degueldre C, Leclercq Y, Phillips C, Luxen A, Vandewalle G, Vogels R, Maquet P, Balteau E (2013) The impact of visual perceptual learning on sleep and local slow-wave initiation. J Neurosci 33:3323–3331. 10.1523/JNEUROSCI.0763-12.2013 23426660 PMC6619511

[B30] Massimini M, Huber R, Ferrarelli F, Hill S, Tononi G (2004) The sleep slow oscillation as a traveling wave. J Neurosci 24:6862–6870. 10.1523/JNEUROSCI.1318-04.2004 15295020 PMC6729597

[B31] Menicucci D, Piarulli A, Debarnot U, d'Ascanio P, Landi A, Gemignani A (2009) Functional structure of spontaneous sleep slow oscillation activity in humans. PLoS One 4:e7601. 10.1371/journal.pone.0007601 19855839 PMC2762602

[B32] Mensen A, Riedner B, Tononi G (2016) Optimizing detection and analysis of slow waves in sleep EEG. J Neurosci Methods 274:1–12. 10.1016/j.jneumeth.2016.09.006 27663980

[B33] Mölle M, Marshall L, Gais S, Born J (2002) Grouping of spindle activity during slow oscillations in human non-rapid eye movement sleep. J Neurosci 22:10941–10947. 10.1523/JNEUROSCI.22-24-10941.2002 12486189 PMC6758415

[B34] Montplaisir J, Nielsen T, Côté J, Boivin D, Rouleau I, Lapierre G (1990) Interhemispheric EEG Coherence before and after partial callosotomy. Clin Electroencephalogr 21:42–47. 10.1177/155005949002100114 2297948

[B35] Murphy M, Riedner BA, Huber R, Massimini M, Ferrarelli F, Tononi G (2009) Source modeling sleep slow waves. Proc Natl Acad Sci U S A 106:1608–1613. 10.1073/pnas.0807933106 19164756 PMC2635823

[B36] Neske GT (2016) The slow oscillation in cortical and thalamic networks: mechanisms and functions. Front Neural Circuits 9:88. 26834569 10.3389/fncir.2015.00088 PMC4712264

[B37] Nielsen T, Montplaisir J, Lassonde M (1993) Decreased interhemispheric EEG coherence during sleep in agenesis of the corpus callosum. Eur Neurol 33:173–176. 10.1159/000116928 8467828

[B38] Piantoni G, Poil S-S, Linkenkaer-Hansen K, Verweij IM, Ramautar JR, Van Someren EJW, Van Der Werf YD (2013) Individual differences in white matter diffusion affect sleep oscillations. J Neurosci 33:227–233. 10.1523/JNEUROSCI.2030-12.2013 23283336 PMC6618630

[B39] Rattenborg NC, Amlaner CJ, Lima SL (2000) Behavioral, neurophysiological and evolutionary perspectives on unihemispheric sleep. Neurosci Biobehav Rev 24:817–842. 10.1016/s0149-7634(00)00039-7 11118608

[B40] Riedner BA, Vyazovskiy VV, Huber R, Massimini M, Esser S, Murphy M, Tononi G (2007) Sleep homeostasis and cortical synchronization: III. A high-density EEG study of sleep slow waves in humans. Sleep 30:1643–1657. 10.1093/sleep/30.12.1643 18246974 PMC2276133

[B41] Sanchez E, El-Khatib H, Arbour C, Bedetti C, Blais H, Marcotte K, Baril A-A, Descoteaux M, Gilbert D, Carrier J, Gosselin N, Sanchez E, Marcotte K, Bedetti C, Baril A-A, Arbour C, Carrier J, Gosselin N, Gilbert D, El-Khatib H, et al (2019) Brain white matter damage and its association with neuronal synchrony during sleep. Brain 142:674–687. 10.1093/brain/awy348 30698667 PMC6391600

[B42] Sanchez-Vives MV, McCormick DA (2000) Cellular and network mechanisms of rhythmic recurrent activity in neocortex. Nat Neurosci 3:1027–1034. 10.1038/79848 11017176

[B43] Schoch SF, Riedner BA, Deoni SC, Huber R, Lebourgeois MK, Kurth S (2018) Across-night dynamics in traveling sleep slow waves throughout childhood. Sleep 41:zsy165 10.1093/sleep/zsy165 PMC6231526 30169809

[B44] Schönemann PH (1966) A generalized solution of the orthogonal procrustes problem. Psychometrika 31:1–10. 10.1007/BF02289451

[B45] Sekimoto M, Kato M, Kajimura N, Watanabe T, Takahashi K, Okuma T (2000) Asymmetric interhemispheric delta waves during all-night sleep in humans. Clin Neurophysiol 111:924–928. 10.1016/s1388-2457(00)00258-3 10802465

[B46] Sekimoto M, Kato M, Watanabe T, Kajimura N, Takahashi K (2007) Reduced frontal asymmetry of delta waves during all-night sleep in schizophrenia. Schizophr Bull 33:1307–1311. 10.1093/schbul/sbl069 17172634 PMC2779871

[B47] Siclari F, Bernardi G, Riedner BA, LaRocque JJ, Benca RM, Tononi G (2014) Two distinct synchronization processes in the transition to sleep: a high-density electroencephalographic study. Sleep 37:1621–1637. 10.5665/sleep.4070 25197810 PMC4173919

[B48] Siclari F, Bernardi G, Cataldi J, Tononi G (2018) Dreaming in NREM sleep: a high-density EEG study of slow waves and spindles. J Neurosci 38:9175–9185. 10.1523/JNEUROSCI.0855-18.2018 30201768 PMC6199409

[B49] Spiess M, Bernardi G, Kurth SS, Ringli M, Wehrle FM, Jenni OG, Huber R, Siclari F, Wehrle FM, Jenni OG, Huber R, Siclari F (2018) How do children fall asleep? A high-density EEG study of slow waves in the transition from wake to sleep. Neuroimage 178:23–35. 10.1016/j.neuroimage.2018.05.024 29758338

[B50] Steriade M, Timofeev I, Grenier F (2001) Natural waking and sleep states: a view from inside neocortical neurons. J Neurophysiol 85:1969–1985. 10.1152/jn.2001.85.5.1969 11353014

[B51] Tamaki M, Bang JW, Watanabe T, Sasaki Y (2016) Night watch in one brain hemisphere during sleep associated with the first-night effect in humans. Curr Biol 26:1190–1194. 10.1016/j.cub.2016.02.063 27112296 PMC4864126

[B52] Tarpley RJ, Ridgway SH (1994) Corpus callosum size in delphinid cetaceans. Brain Behav Evol 44:156–165. 10.1159/000113587 7987664

[B53] Timofeev I, Steriade M (1996) Low-frequency rhythms in the thalamus of intact-cortex and decorticated cats. J Neurophysiol 76:4152–4168. 10.1152/jn.1996.76.6.4152 8985908

[B54] Timofeev I, Grenier F, Bazhenov M, Sejnowski TJ, Steriade M (2000) Origin of slow cortical oscillations in deafferented cortical slabs. Cereb Cortex 10:1185–1199. 10.1093/cercor/10.12.1185 11073868

[B55] Timofeev I, Sejnowski TJ, Bazhenov M, Chauvette S, Grand LB (2013) Age dependency of trauma-induced neocortical epileptogenesis. Front Cell Neurosci 7:154. 10.3389/fncel.2013.00154 24065884 PMC3776140

[B56] Tononi G, Cirelli C (2014) Sleep and the price of plasticity: from synaptic and cellular homeostasis to memory consolidation and integration. Neuron 81:12–34. 10.1016/j.neuron.2013.12.025 24411729 PMC3921176

[B57] Topolnik L, Steriade M, Timofeev I (2003) Partial cortical deafferentation promotes development of paroxysmal activity. Cereb Cortex 13:883–893. 10.1093/cercor/13.8.883 12853375

[B58] Vantomme G, Osorio-Forero A, Lüthi A, Fernandez L (2019) Regulation of local sleep by the thalamic reticular nucleus. Front Neurosci 13:576. 31231186 10.3389/fnins.2019.00576 PMC6560175

[B59] Vyazovskiy VV, Borbély AA, Tobler I (2002) Interhemispheric sleep EEG asymmetry in the rat is enhanced by sleep deprivation. J Neurophysiol 88:2280–2286. 10.1152/jn.00304.2002 12424269

[B60] Vyazovskiy VV, Riedner BA, Cirelli C, Tononi G (2007) Sleep homeostasis and cortical synchronization: II. A local field potential study of sleep slow waves in the rat. Sleep 30:1631–1642. 10.1093/sleep/30.12.1631 18246973 PMC2276140

[B61] Xie L, Kang H, Xu Q, Chen MJ, Liao Y, Thiyagarajan M, O'Donnell J, Christensen DJ, Nicholson C, Iliff JJ, Takano T, Deane R, Nedergaard M (2013) Sleep drives metabolite clearance from the adult brain. Science 342:373–377. 10.1126/science.1241224 24136970 PMC3880190

